# Circular RNA profiling reveals an abundant circHIPK3 that regulates cell growth by sponging multiple miRNAs

**DOI:** 10.1038/ncomms11215

**Published:** 2016-04-06

**Authors:** Qiupeng Zheng, Chunyang Bao, Weijie Guo, Shuyi Li, Jie Chen, Bing Chen, Yanting Luo, Dongbin Lyu, Yan Li, Guohai Shi, Linhui Liang, Jianren Gu, Xianghuo He, Shenglin Huang

**Affiliations:** 1 Fudan University Shanghai Cancer Center and Institutes of Biomedical Sciences, Shanghai Medical College, Fudan University; Department of Oncology, Shanghai Medical College, Fudan University, Shanghai 200032, China; 2 State Key Laboratory of Oncogenes and Related Genes, Shanghai Cancer Institute, Renji Hospital, Shanghai Jiao Tong University School of Medicine, Shanghai 200032, China

## Abstract

Circular RNAs (circRNAs) represent a class of widespread and diverse endogenous RNAs that may regulate gene expression in eukaryotes. However, the regulation and function of human circRNAs remain largely unknown. Here we generate ribosomal-depleted RNA sequencing data from six normal tissues and seven cancers, and detect at least 27,000 circRNA candidates. Many of these circRNAs are differently expressed between the normal and cancerous tissues. We further characterize one abundant circRNA derived from Exon2 of the *HIPK3* gene, termed circHIPK3. The silencing of circHIPK3 but not *HIPK3* mRNA significantly inhibits human cell growth. Via a luciferase screening assay, circHIPK3 is observed to sponge to 9 miRNAs with 18 potential binding sites. Specifically, we show that circHIPK3 directly binds to miR-124 and inhibits miR-124 activity. Our results provide evidence that circular RNA produced from precursor mRNA may have a regulatory role in human cells.

## Abstract (web summary)

Circular RNAs are formed from exon back-splicing, the significance of these endogenous RNAs is beginning to be unraveled. Here, the authors identify thousands of circular RNAs differentially expressed between normal and cancer tissues and show that an abundant circular RNA generated from *HIPK3* regulates cell growth.

## Graphical abstract

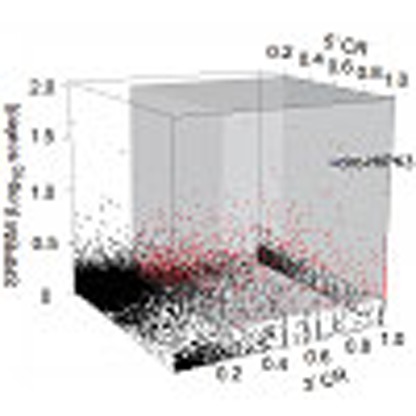

Circular RNAs from back-spliced exons (circRNAs) are recently identified as a naturally occurring family of noncoding RNAs that is highly represented in the eukaryotic transcriptome^1^,^2^. The formation of circRNAs had occasionally been identified more than 20 years ago from a few transcribed genes^3^,^4^,^5^. Nevertheless, these species had generally been considered to be of low abundance and likely representing errors in splicing. Thus, their widespread and substantial presence within transcriptomes has only recently been demonstrated via the high-throughput sequencing and novel computational approaches^1^,^6^,^7^,^8^. Specifically, a large number of circRNAs have been successfully identified in various cell lines and across different species^9^,^10^,^11^,^12^,^13^.

circRNAs are characterized by covalently closed loop structures with neither 5′ to 3′ polarity nor a polyadenylated tail. They are highly stable *in vivo* compared with their linear counterparts, and are predominantly in the cytoplasm and can be sorted into exosomes^14^. Two mechanisms, ‘exon skipping' and ‘direct back-splicing', have been proposed to form mammalian exonic circRNA^1^,^6^,^7^. Exon skipping leads to a lariat whose restricted structure promotes circularization, whereas direct back-splicing refers to the pairing of a downstream splice donor with an unspliced upstream splice acceptor, which results in the circularization of the intervening RNA. Both mechanisms involve back-splicing being formed by the canonical spliceosome^15^. Recent studies have shown that exon circularization is facilitated by complementary sequences^16^,^17^ and regulated by specific protein factors^18^,^19^,^20^. It is becoming increasingly evident that circRNAs are not simply by-products of mis-splicing or splicing errors, rather, they are the products of regulated back-splicing with distinct sets of cis-elements and/or trans-factors^21^. Accordingly, many circRNAs have been found to be upregulated during mouse neural development and human epithelial–mesenchymal transition^10^,^20^. Recently, circRNAs have been shown to act as microRNA (miRNA) sponges to regulate gene expression^8^,^22^. Specifically, the circRNA ciRS-7 (also termed CDR1as), which harbours more than 70 conventional miR-7-binding sites, has been identified as a miRNA inhibitor. However, only a few such circRNAs contain multiple binding sites to trap one particular miRNA^11^, and the function of circRNA remains largely unknown.

In humans, circRNAs have been characterized in several cell lines and brain tissue^9^,^10^,^11^,^12^. In this study, we generated ribominus RNA sequencing data from six human normal tissues and seven human cancers, and identified ∼27,000 circRNA candidates (at least two unique back-spliced reads). Analysis of these circRNAs revealed that there is often a predominately expressed circRNA isoform from a given gene locus and a number of circRNAs are highly abundant. We further characterize one abundant circRNA produced from the *HIPK3* gene, termed circHIPK3. The formation of circHIPK3 is due to the long intronic complementary repeat elements. Importantly, we found that circHIPK3 RNA, but not *HIPK3* mRNA, functions as a cell growth modulator in human cells. We further performed a luciferase screening and observed that circHIPK3 could bind to multiple miRNAs, including a well-known tumor suppressor miRNA miR-124. Our findings indicate that protein-coding exons may exert additional regulatory functions when expressed within circRNAs in human cells.

## Results

### Profiling of circRNAs in human normal and cancerous tissues

**Figure 1:**
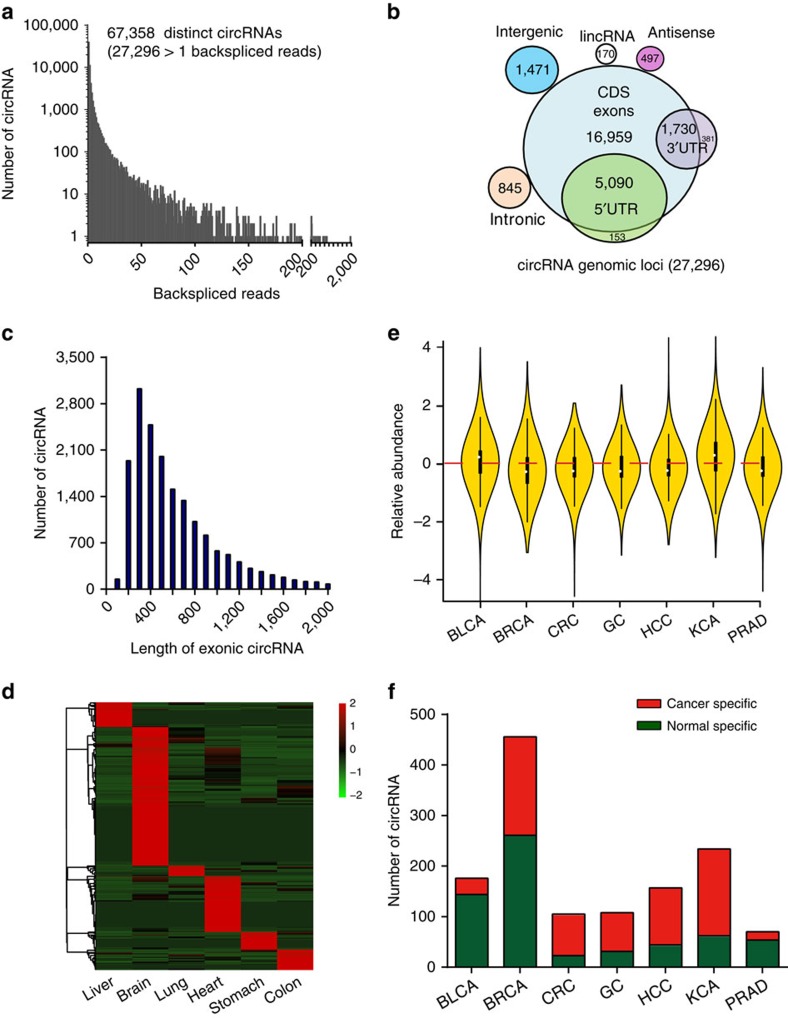
Profiling of circular RNAs in human normal and cancerous tissues. (**a**) The number of circRNAs and back-spliced reads identified in six human normal tissues and seven human cancerous tissues. (**b**) Genomic origin of human circRNAs. (**c**) The length distribution for exonic circRNAs (*n*=20,533, only known spliced length was considered). (**d**) Clustered heatmap for tissue-specific circRNAs from six human normal tissues, with rows representing circRNAs and columns representing tissues. The circRNAs were classified according to the Pearson correlation. The numerical data represented log10-transformed mean SRPBM of two replicates. (**e**) Violin plot of relative abundance of circRNAs in seven cancer tissues compared with the paired normal tissues. Data are expressed as the log_2_ foldchange of SRPBM. The white dot represents the median. (**f**) Numbers of specific circRNAs identified in seven cancerous and matched normal tissues. Cancer-specific circRNAs are shown in red. Normal specific circRNAs are shown in green.

First, we characterized circRNA transcripts using RNA-sequencing (RNA-seq) analyses of ribosomal RNA-depleted total RNA from six normal tissues (brain, colon, heart, liver, lung and stomach) and seven cancerous tissues (bladder urothelial carcinoma (BLCA), breast cancer, colorectal cancer (CRC), hepacellular carcinoma (HCC), gastric cancer (GC), kidney clear cell carcinoma (KCA) and prostate adenocarcinoma (PRAD)). Each sample was sequenced on an Illumina HiSeq yielding ∼60 million reads, which were mapped to the human reference genome (GRCh37/hg19) by TopHat2 (ref. 23). A detailed summary for each sample is provided in Supplementary Table 1. A computational pipeline based on the anchor alignment of unmapped reads was used to identify circRNAs without relying on gene annotations^8^ (Supplementary Fig. 1). In total, 67,358 distinct circRNA candidates were found in these tissues and 27,296 of these circRNAs contained at least two unique back-spliced reads (Fig. 1a, Supplementary Data 1). Compared with previously published databases obtained from circBase^9^ (92,061 human circRNAs) and a most recent study^10^ (65,731 human circRNAs identified mainly from human brain tissues), we found that there are 19,071 overlapped circRNAs and 8,225 novel circRNAs identified in our study (Supplementary Fig. 2 and Supplementary Data 1). Notably, there are totally 148,701 unique human circRNA candidates from all the studies, indicating that circRNAs may contain one of the largest RNA families in human transcription.

We annotated these circRNA candidates using the RefSeq database^24^. More than 80% of the circRNAs consisted of protein-coding exons, whereas smaller fractions aligned with introns, long noncoding RNAs, unannotated regions of the genome and antisense regions to known transcripts (Fig. 1b). The length of most exonic circRNAs (*n*=20,553; only known splice lengths without introns) was less than 1,500 nucleotide (nt), and the median length was ∼500 nt (Fig. 1c, Supplementary Data 1). We normalized the back-spliced reads (support for circRNA) by read length and number of mapped reads (spliced reads per billion mapping, denoted as SRPBM), which permits quantitative comparisons between back splicing from different RNA-seq data^7^. The expression analysis of these circRNA transcripts revealed that numerous circRNAs seem to be specifically expressed across various tissues (Fig. 1d, minimum value of specificity score 0.5 and minimum level of mean+2s.d., Methods section). Analogously, the profile of circRNAs (SRPBM>1) in cancer often differs from that of normal tissue (Fig. 1e). The differences of circRNAs expression were calculated using Wilcoxon rank-sum test by comparing the cancerous sample with matched normal sample for each tissue. We found that circRNAs are significantly downregulated in BRCA (*P*=4.71e–32), CRC (*P*=9.50e–35), GC (*P*=5.09e–10), HCC (*P*=1.86e–32) and PRAD (*P*=1.52e–08) tumours, and upregulated in BLCA (*P*=1.13e–09) and KCA (*P*=3.65e–79). However, the parent genes are significantly upregulated in BLCA (*P*=8.15e–49), BRCA (*P*=5.07e–204), GC (*P*=5.49e–206) and HCC (*P*=1.47e–32) and downregulated in CRC (*P*=1.94e–4) and KCA (*P*=4.51e–07), but unchanged in PRAD (*P*=0.4320). Only BLCA showed the similar expression patterns, indicating that circRNAs and/or parent genes are post-transcriptionally regulated. Moreover, a number of circRNAs seem to be specifically expressed in cancer and matched normal tissues (Fig. 1f, at least four reads as the specificity cut-off, Methods section).

To verify that the back-spliced events were indicative of true circular, and not linear, trans-splicing products, we examined the physical properties of these products. Outward-facing primers were designed against 36 commonly expressed circRNA transcript candidates (Supplementary Table 2). Each primer pair amplified a single, distinct product of the expected size from HEK-293T cDNA (Supplementary Fig. 3A). The enrichment of all 36 back-spliced events was apparent following RNase R treatment, whereas the abundance of linear RNAs decreased (Supplementary Fig. 3B). We also validated 20 novel circRNAs (out of 26 circRNA candidates) in original tissue samples by quantitative reverse transcription-PCR(qRT–PCR) with RNase R treatment and RT–PCR with Sanger sequencing (Supplementary Fig. 4).

### The characteristics of circRNA abundance in human cells

**Figure 2:**
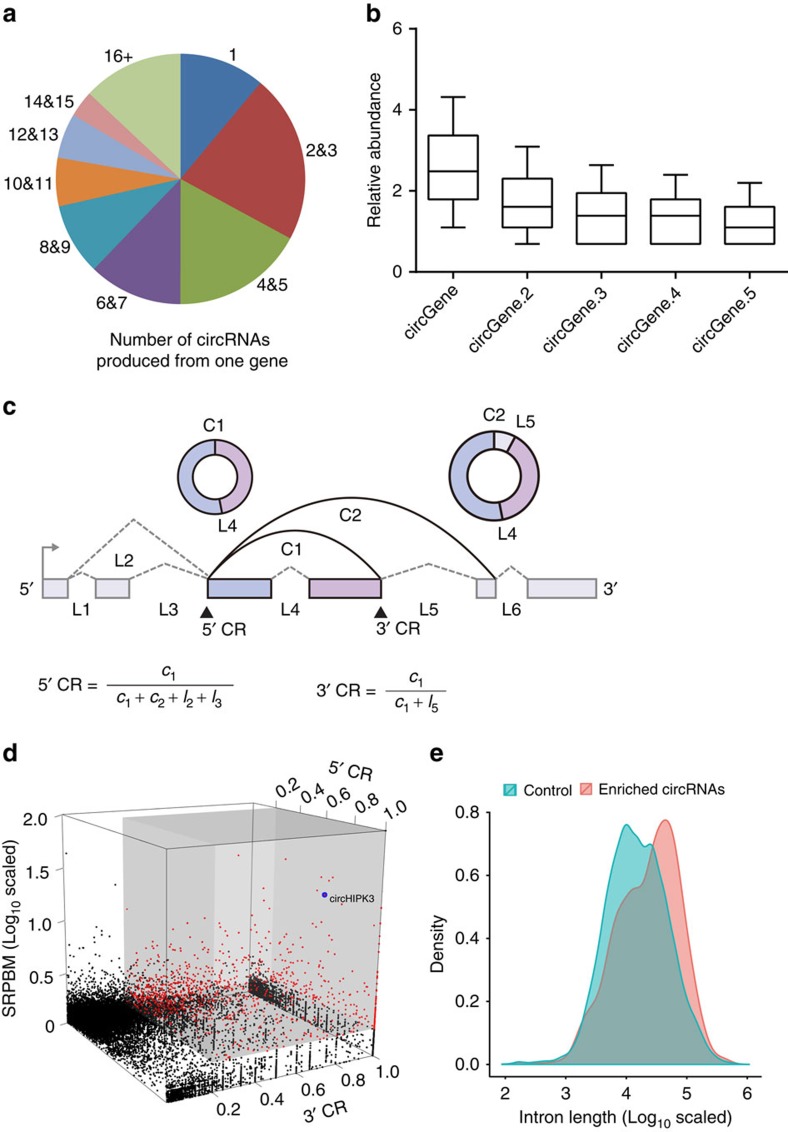
The characteristics of circular RNA abundance in human cells. (**a**) Number of circRNAs produced from one gene (20,530 circRNAs from 5,955 host genes). (**b**) The box plots describe the comparison of the levels of most abundantly expressed circRNA isoform (circGene) and other circRNAs (circGene.x) from one gene locus (*n*=3,687). The ends of the boxes define the 25th and 75th centiles, a line indicates the median, and bars define the 5th and 95th centiles. The first five circRNAs were presented. (**c**) Schematic illustration of the methodology to estimate either the circular ratio at the 5′ end (5′ CR) or at 3′ end (3′ CR) for circRNAs. *C*_i_ and *c*_i_ represent the back-spliced junctions (support for circRNA) and number of reads spanning these junctions, respectively; *L*_i_ and *l*_i_ represented linear spliced junctions and the number of reads spanning these junctions, respectively. Solid squares, exons; broken lines, linear spliced junctions; arc lines, back-spliced junctions. (**d**) Multidimensional scaling screen for highly abundant circRNAs (*n*=27,293, three outlines were not shown). Red dots and black dots represented highly abundant circRNAs and low-abundance circRNAs, respectively. circHIPK3 is highlighted in blue. The cut-off for highly abundant circRNAs is shown in grey. The pseudocount of 1 was added to SRPBM to avoid log_10_-transform issues. Thus, the cut-off of log10-transformed SRPBM is 0.3010. (**e**) A density plot of the flanking intron length of highly abundant circRNAs (red) and low-abundance circRNAs (green).

Analysis of the number of circRNAs from their host genes revealed that one gene could produce multiple circRNAs (Fig. 2a, 20,530 circRNAs from 5,955 host genes), which is consistent with previous report^16^. A striking example is the oncogene PTK2 that may generate 47 distinct circRNAs (at least two unique back-spliced reads). We further investigated the abundance of the circRNAs within one gene locus, and found that about 50% of the host genes (1,835/3,687, single circRNA from one gene not included) produced a significantly higher expressed circRNAs (at least twofold higher than other circRNAs, Fig. 2b). This result indicates that there is often a predominantly expressed circRNA isoform from one gene locus. Anchor alignment also allows us to quantify the abundance of each circRNA with respect to its alternative linear isoform in the ribosomal RNA-depleted RNA-seq data (Fig. 2c and Methods section). For each of these circRNAs, we estimated the circular ratio (CR) of a circRNA at the 5′ end or 3′ end according to the number of reads spanning the back-spliced junctions and the number of reads spanning the linearly spliced junctions. The CR for these sites considerably varied, and linearly spliced products were absent in some cases (Fig. 2d and Supplementary Data 2). When using a stringency of CR and SRPBM cut-off (5′ CR>0.2; 3′ CR>0.2; SRPBM>1), we observed 990 high-abundance circRNAs, many of which were more highly expressed than their linear isoforms (Fig. 2d). Analysing these circRNAs revealed that flanking introns were markedly longer than other circRNAs (Fig. 2e, *P*=1.20e–21, Mann–Whitney *U*-test).

**Figure 3:**
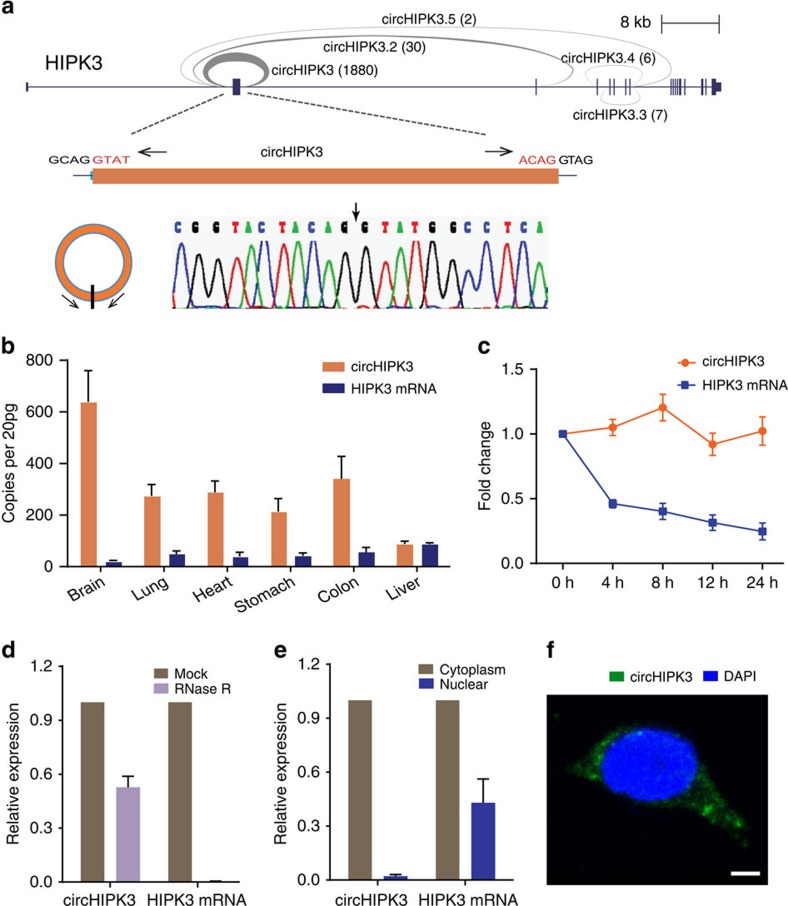
Characterization of circHIPK3 RNA in human cells. (**a**) The genomic loci of five circRNAs in *HIPK3* gene. The supported unique reads were presented. The expression of circHIPK3 was validated by RT–PCR followed by sanger sequencing. Arrows represent divergent primers binding to the genome region of circHIPK3. (**b**) Absolute quantification for circHIPK3 and *HIPK3* mRNA in six human normal tissues. (**c**) qRT–PCR for the abundance of circHIPK3 and *HIPK3* mRNA in HeLa cells treated with Actinomycin D at the indicated time points. (**d**) qRT–PCR for the abundance of circHIPK3 and *HIPK3* mRNA in HeLa cells treated with RNase R. The amount of circHIPK3 and *HIPK3* mRNA were normalized to the value measured in the mock treatment. (**e**) qRT–PCR data indicating the abundance of circHIPK3 and *HIPK3* mRNA in either the cytoplasm or nucleus of HeLa cells. The amounts of circHIPK3 and *HIPK3* mRNA were normalized to the value measured in the cytoplasm. Data in (**c**–**e**) are the means±s.e.m. of three experiments. (**f**) RNA fluorescence *in situ* hybridization for circHIPK3. Nuclei were stained with 4,6-diamidino-2-phenylindole (DAPI). Scale bar, 5 μm.

We further noted that one of these circRNAs, derived from the *HIPK3* gene Exon2 (termed circHIPK3), was particularly abundant and featured a high back-spliced ratio (Fig. 2d, blue dot). The high abundance of circHIPK3 was also observed by previous reports^7^,^17^. Besides circHIPK3, there are another four circRNA isoforms identified in our study in *HIPK3* gene locus (We termed them as circHIPK3.2, circHIPK3.3, circHIPK3.4, circHIPK3.5, respectively; Fig. 3a). circHIPK3 is the predominant circRNA isoform as evidence from the high-supported back-spliced unique reads (circHIPK3, 1880; circHIPK3.2, 30; circHIPK3.3, 7; circHIPK3.4, 6; circHIPK3.5, 2). The genomic structure shows that circHIPK3 contains a large second exon (1,099 bp) from the *HIPK3* gene flanked by long introns on either side (Fig. 3a). The distinct product of the expected size was amplified using outward-facing primers and confirmed by Sanger sequencing (Fig. 3a). The circular expression levels were quantified by qRT–PCR with divergent primers calibrated by standard curves. Consistent with the RNA-seq results, circHIPK3 was significantly more abundant in various tissues (except for liver tissue) than the linear form as indicated by qRT–PCR analysis (Fig. 3b). circHIPK3 is commonly expressed in various tissues (100–600 copies per cell, assuming 20 pg RNA per cell) and particularly enriched in the brain (Fig. 3b). We then investigated the stability and localization of this circRNA in HeLa cells. Total RNA was harvested at the indicated time points after treatment with Actinomycin D, an inhibitor of transcription. An analysis of circHIPK3 and *HIPK3* mRNA revealed that the circRNA isoform was highly stable, with transcript half-life exceeding 24 h, whereas the associated linear transcript exhibited half-life of <4 h (Fig. 3c). Resistance to digestion with RNase R exonuclease further confirmed that this RNA specie is circular in form (Fig. 3d). qRT–PCR analysis of nuclear and cytoplasmic circHIPK3 RNA and fluorescence *in situ* hybridization (FISH) against circHIPK3 demonstrated that the circular form of *HIPK3* preferentially localized in the cytoplasm (Fig. 3e,f). Taken together, our results show that circHIPK3 is an abundant and stable circRNA expressed in different human cells.

### The formation of circHIPK3 from *HIPK3* exon2

**Figure 4:**
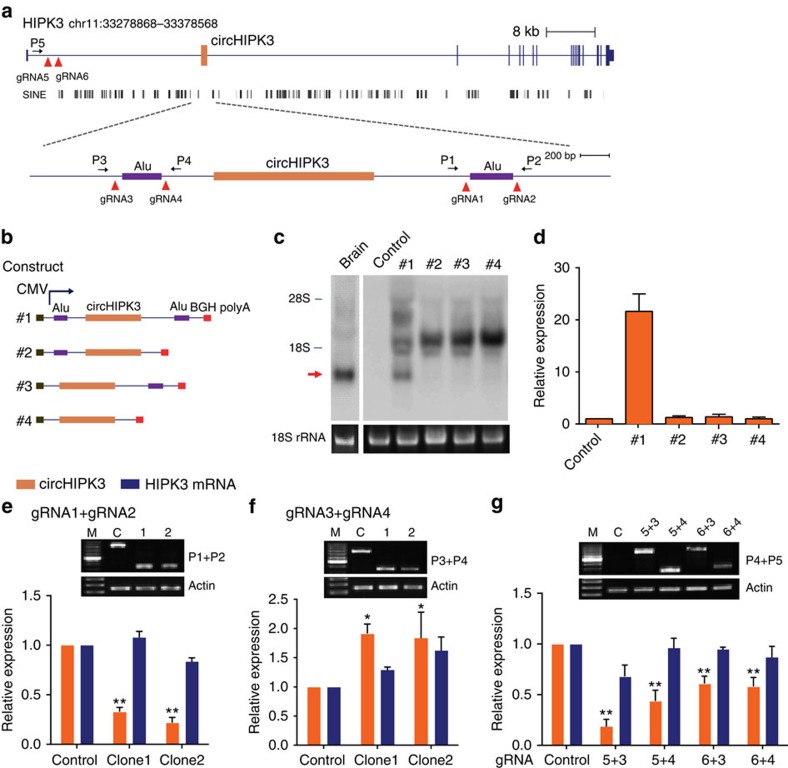
circHIPK3 is derived from *HIPK3* exon2 due to long flanking introns. (**a**) Schematics showing that the genomic regions of HIPK3 Exon2 contain flanking Alu repeats and long introns. Alu elements and long introns were deleted using CRISPR/Cas9 systems. Primers flanking the gRNA recognition were used to detect deletions. (**b**) Schematic diagram of circHIPK3 expression vectors with various genomic sequences for circHIPK3 recapitulation (#1–4). The genomic region of circHIPK3 was cloned into the pcDNA3.0 vector with the upstream and downstream intron sequences, which included Alu elements (#1). Deletions were introduced into the HIPK3 expression plasmid. (#2–4). (**c**,**d**) Northern blot and qRT–PCR for circHIPK3 in cells transfected with the expression plasmids (#1–4). (**e**,**f**) Normal PCR and qRT–PCR data for CRISPR/Cas9-mediated genomic deletions in HEK-293 T cells. Two pairs of gRNAs (gRNA1+gRNA2, gRNA3+gRNA4) were used to mediate the deletion of the proximal Alu elements. M: 100 bp DNA ladder. (**g**) Normal PCR and qRT–PCR data for large deletion mediated by four sets of paired gRNAs. The primer site was designed outside the deleted region. For the control gRNA, the expected product was not shown because of too large PCR product. For the loading control, a genomic region from Actin gene loci was amplifed in **e**–**g**. Data in **d**–**g** are the means±s.e.m. of three experiments. *P<0.05, **P<0.01 (Student's *t*-test).

We next investigated the mechanism by which circHIPK3 is formed. An analysis of the flanking introns of *HIPK3* Exon2 showed highly complementary Alu repeats with 28 short interspersed elements in the intron upstream of *HIPK3* Exon2 and 51 short interspersed elements downstream from Exon3 (Fig. 4a). Among these repeats, two primate-specific Alu elements in an inverted orientation immediately flank the *HIPK3* circularizing exon (Fig. 4a). The inverted repeated Alu elements (IRAlus) are highly reverse complementary (83% identity over 269 nt; Supplementary Fig. 5). The relatively short introns may be sufficient to support circularization as suggested by a recent report^17^. Thus, a 3,126-nt region of the *HIPK3* pre-mRNA, spanning from 1,039 nt upstream of Exon2 to 988 nt downstream of Exon3, was cloned into the pcDNA3 expression vector (Fig. 4b). The expected circRNA production was detected by northern blot and qRT–PCR analysis after transfection with the expression vector (Fig. 4c,d). Consistent with previous result^17^, circularization was not observed when one or both Alu elements were deleted (Fig. 4c,d), indicating that the two inverted repeat elements are indispensable to the formation of circHIPK3 in the expression vector.

We further used CRISPR/Cas9 technology to delete the repeated sequences within cells. Two gRNAs to the boundaries of the Alu sequence were designed (Fig. 4a). Each gRNA pair was co-transfected with the Cas9 expression vector to delete the repeated sequences in HEK-293T cells. The efficiencies of targeted deletion were determined by PCR analyses using primers flanking the targeted regions. Wild-type and truncated genomic fragments were resolved by gel electrophoresis. We observed that circularization was inhibited when the downstream Alu sequence was deleted (Fig. 4e). Surprisingly, the deletion of the upstream Alu sequence did not decrease the formation of circHIPK3, rather, it slight increased the formation of this RNA (Fig. 4f). Because there are many Alu elements upstream of the circularized exon, we hypothesized that the other Alu elements within the intron may facilitate circularization. To test this hypothesis, we further deleted the large upstream intron using four sets of paired gRNAs (Fig. 4a). The result showed that circHIPK3 was significantly downregulated on the deletion of the intron (Fig. 4g). However, this deletion did not significantly influence the expression of *HIPK3* mRNA (Fig. 4g, blue bar). Therefore, the deletion affected only back splicing, but not canonical splicing. These results suggest that the long flanking introns with complementary Alu repeats are required for the formation of circHIPK3.

### Silencing of circHIPK3 inhibits human cell proliferation

**Figure 5:**
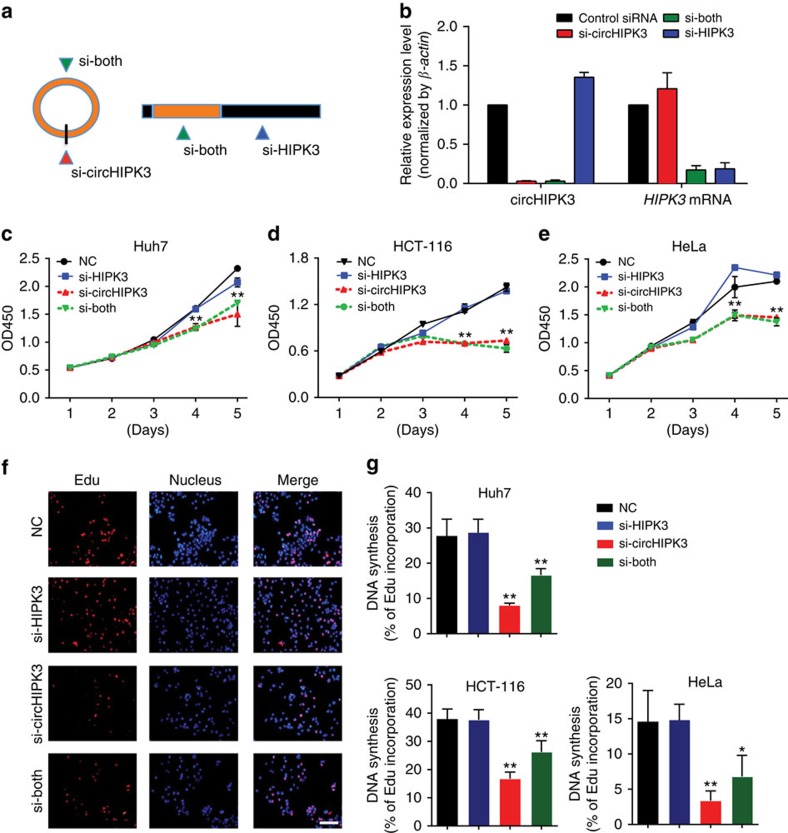
Silencing of circHIPK3 RNA inhibits human cell proliferation. (**a**) Schematic illustration showing three targeted siRNAs. Si-HIPK3 targets the *HIPK3* linear transcript, si-circHIPK3 targets the back-splice junction of circHIPK3, and si-both targets both the linear and circular species. (**b**) qRT–PCR for circHIPK3 and *HIPK3* mRNA in HEK-293 T cells treated with three siRNAs as described above. Data are the means±s.e.m. of three experiments. (**c**–**e**) Proliferation of HuH-7, HCT-116 and HeLa cells transfected with the above three siRNAs assessed using a CCK-8 kit at the indicated days. Data in **c**–**e** are the means±s.e.m. of three experiments. (**f**) DNA synthesis assessed using an EdU (5-ethynyl-2'-deoxyuridine) assay in HuH-7 cells transfected with the above three siRNAs for 48 h. Cells were fluorescently stained with EdU (red). Nuclei were stained with DAPI (blue). Micrographs represent at least three experiments. Scale bar, 200 μm. (**g**) Quantitative EdU assay data from **f** and Supplementary Fig. 7. Data in **d**–**g** are the means±s.e.m. of three experiments. *P<0.05, **P<0.01 (Student's *t*-test).

We noted that circHIPK3 was significantly upregulated in liver cancer compared with matched normal tissues (Supplementary Fig. 6), which prompted us to investigate the function of circHIPK3. Because circRNA can be targeted by small interference RNA^7^, we used RNA interference to silence the expression of both circHIPK3 and *HIPK3* mRNA. We designed three small interfering RNAs (siRNAs): one siRNA targeting the backsplice sequence, another siRNA targeting sequence only in the linear transcript, and a third siRNA targeting sequence in a circularized exon shared by both linear and circular species (Fig. 5a). A nonspecific control siRNA sequence was also employed. As expected, siRNA directed against the backsplice sequence knocked down only the circular transcript and did not affect the expression of linear species (Fig. 5b). siRNA targeted to exonic sequences shared by both the linear and circular species effectively knocked down both transcripts (Fig. 5b). A subsequent cell proliferation assay indicated that the downregulation of circHIPK3, but not *HIPK3* mRNA, significantly suppressed cell growth in different cell types (Fig. 5c-e). An EdU incorporation assay also revealed that the proliferation of various human cells was impaired on the knockdown of circHIPK3 expression (Fig. 5f,g and Supplementary Fig. 7). Consistent with this result from the siRNA knockdown experiments, we observed that cell proliferation was significantly suppressed on the knockdown of circHIPK3 expression in HEK-293 T cells via CRISPR/Cas9 technology (Supplementary Fig. 8). These findings revealed that circHIPK3 may function to modulate the growth of human cells.

### circHIPK3 serves as a sponge for multiple miRNAs

**Figure 6:**
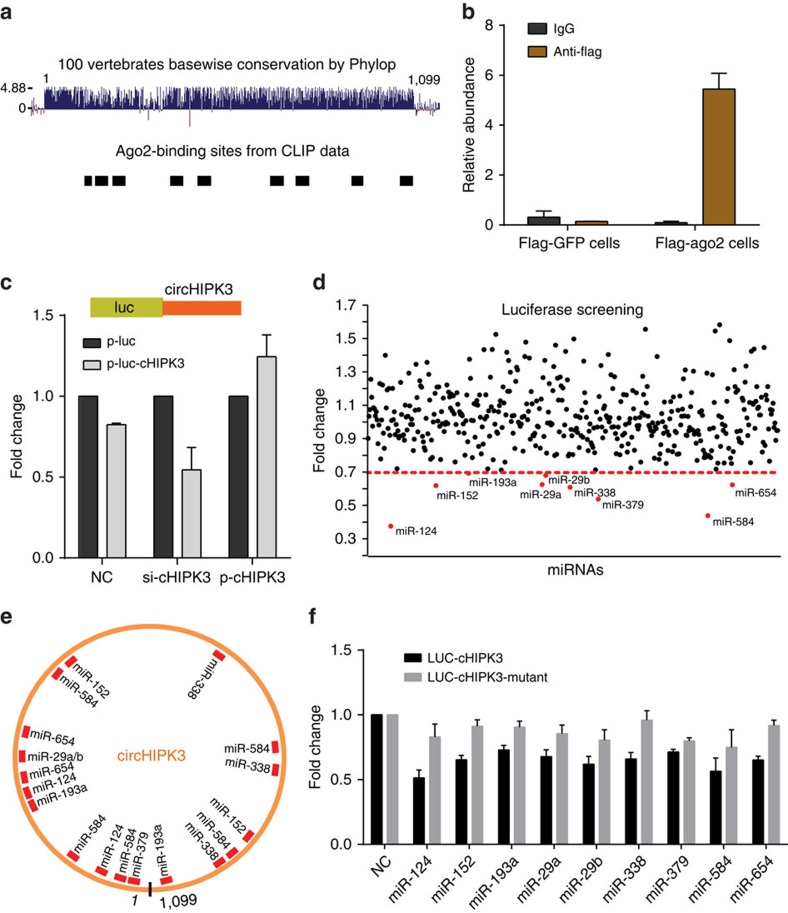
circHIPK3 serves as a sponge for multiple miRNAs in human cells. (**a**) Schematic illustration showing that the conservation across 100 vertebrate species and AGO2 binding sites in circHIPK3 genomic region. (**b**) Ago2 RNA immunoprecipitation (RIP) assay for the amount of circHIPK3 in HEK-293 T cells stably expressing Flag-AGO2 or Flag-GFP. Data are the means±s.e.m. of three experiments. (**c**) The entire circHIPK3 sequence (red) was cloned into the downstream region of the luciferase gene, denoted LUC-cHIPK3. Luciferase reporter assay for the luciferase activity of LUC-cHIPK3 in HEK-293 T cells co-transfected with siRNA against circHIPK3 or circHIPK3 expressing vector. Data are the means±s.e.m. of three experiments. (**d**) Luciferase reporter assay for the luciferase activity of LUC-cHIPK3 in HEK-293 T cells transfected with a library of 424 miRNA mimics to identify miRNAs that were able to bind to the circHIPK3 sequence. Nine miRNAs that inhibited luciferase activity by 30% are indicated by red dots. (**e**) A schematic drawing showing the putative binding sites of the miRNAs associated with circHIPK3. (**f**) Luciferase reporter assay for the luciferase activity of LUC-cHIPK3 or LUC-cHIPK3-mutant in HEK-293 T cells co-transfected with miRNA mimics. Data are the means±s.e.m. of three experiments.

Given that circRNA has been shown to act as miRNA sponge and circHIPK3 is abundant and stable in the cytoplasm, we next investigated the ability of circHIPK3 to bind to miRNAs. An analysis of available online AGO2 immunoprecipitation followed by high-throughput sequencing data from doRiNA^25^ revealed a high degree of AGO2 occupancy in the region of circHIPK3, which is highly conserved across several vertebrate species (Fig. 6a). To validate this result, we conducted RNA immunoprecipitation (RIP) for AGO2 in HEK-293 T cells stably expressing Flag-AGO2 or Flag-GFP, and observed that endogenous circHIPK3 pulled-down from Flag-AGO2 cells was specifically enriched by qRT–PCR analysis (Fig. 6b). We further constructed a circHIPK3 fragment and inserted it immediately downstream of the luciferase reporter gene (LUC+circHIPK3). We hypothesized that circHIPK3-associated miRNAs may potentially inhibit the luciferase activity, presumably via the miRNA-mediated activation of deadenylation and subsequent exonucleolytic degradation. Supporting this hypothesis, we observed that inclusion of the circHIPK3 sequence in the 3′-untranslated region (UTR; without any other cellular perturbations) causes downregulation of luciferase activity (Fig. 6c). Subsequently, knockdown of the endogenous circHIPK3 further decreases the luciferase activity (from the LUC+circHIPK3 plasmid). In contrast, over-expressing circHIPK3 increases the luciferase activity (Fig. 6c). These results suggest that circHIPK3 may serve as a binding platform for AGO2 and miRNAs.

To identify the miRNAs that bind to circHIPK3, we performed a luciferase screening for a miRNA library. Each miRNA mimic was co-transfected with the luciferase reporters into HEK-293 T cells. Compared with the control RNA, 9 miRNAs (miR-124, miR-152, miR-193a, miR-29a, miR-29b, miR-338, miR-379, miR-584 and miR-654) out of the 424 miRNAs were able to reduce the luciferase reporter activities by at least 30% (Fig. 6d). These miRNAs could not significantly decrease circHIPK3 level by qRT–PCR analysis (Supplementary Fig. 9), suggesting that circHIPK3 may not be digested by these miRNAs. Using the TargetScan and PicTar miRNA prediction programs^26^,^27^, these miRNAs were found to contain at least one binding site (18 in total) for the circHIPK3 region (Fig. 6e). We further mutated each miRNA target site from the luciferase reporter with inclusion of the circHIPK3 sequence in the 3′-UTR and circHIPK3 expressing vector, respectively. We found that transfection of the miRNA has no significant effect on luciferase activity when the corresponding target sites were mutated from the luciferase reporter (Fig. 6f). In addition, transfecting a mutant circHIPK3 circle that lacks a given miRNA-binding site is unable to rescue luciferase activities (Supplementary Fig. 10). These results suggest that circHIPK3 may function as a sponge to these miRNAs.

**Figure 7:**
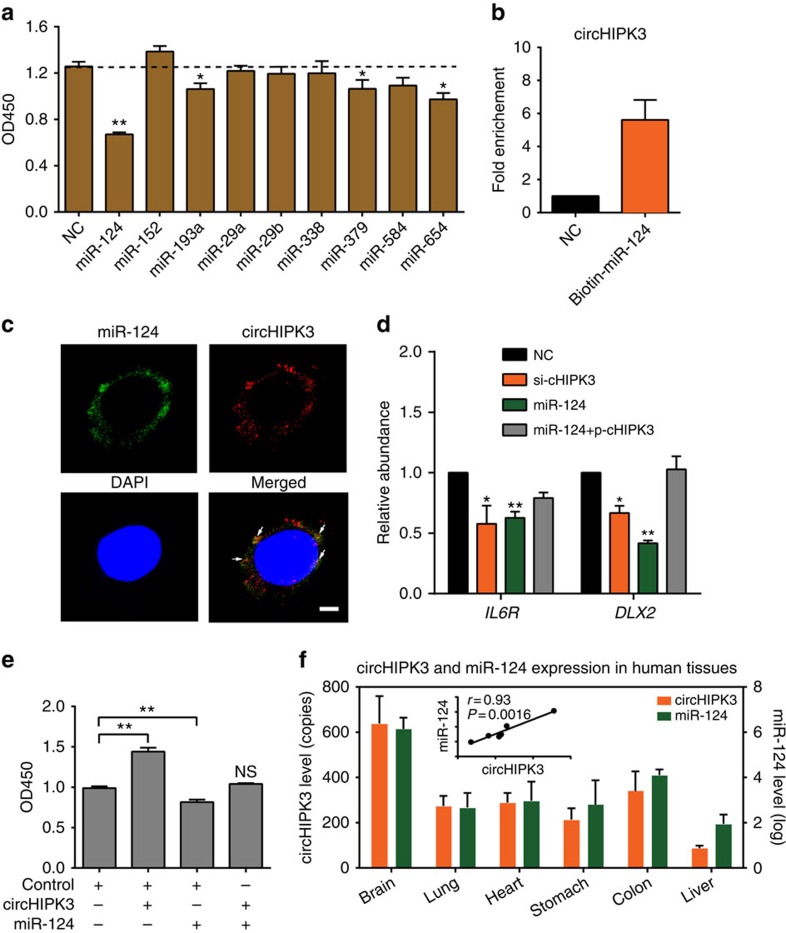
circHIPK3 sponges with miR-124 and inhibits its activity. (**a**) Proliferation assessed using a CCK-8 kit in HEK-293 T cells transfected with nine miRNA mimics or control RNA (20 nM). (**b**) qRT–PCR analysis of circHIPK3 level in the streptavidin captured fractions from the HEK-293 T cell lysates after transfection with 3′-end biotinylated miR-124 or control RNA (NC). (**c**) Co-localization between miR-124 and circHIPK3 was observed (arrowheads) by RNA *in situ* hybridization in HeLa cells after co-transfection with circHIPK3 and miR-124 expressing vectors. Nuclei were stained with DAPI. Scale bar, 5μm. (**d**) qRT–PCR analysis of *IL6R* and *DLX2* expression in HEK-293 T cells after transfected with si-cHIPK3, miR-124 mimics or miR-124 with circHIPK3 expressing vector (p-cHIPK3). (**e**) Proliferation assessed using a CCK-8 kit in cells transfected with circHIPK3 or miR-124 (10 nM) as indicated. Data in **a**,**b**,**d** are the means±s.e.m. of three experiments. (**f**) qRT–PCR for the abundance of circHIPK3 relative to *ACTB* and miR-124 relative RNU6B in six human normal tissues. The correlation between circHIPK3 and miR-124 is also shown. *P<0.05, **P<0.01 (Student's *t*-test).

Notably, all nine miRNAs that found to be sponged by circHIPK3 have been reported to serve as growth-suppressive miRNAs in different cell contexts^28^,^29^,^30^,^31^,^32^,^33^,^34^. We also transfected individual miRNA mimics into HEK-293 T cells. A cell proliferation assay revealed that four of the miRNAs (miR-124, miR-193, miR-379 and miR-654) could significantly inhibit HEK-293 T cell growth, and miR-124 exerted the most striking effect (Fig. 7a). Using a biotin-coupled miR-124 mimic, we observed a more than fivefold enrichment of circHIPK3 in the miR-124-captured fraction compared with the negative control (Fig. 7b). The co-localization experiments are consistent with circHIPK3 interacting with miR-124 (Fig. 7c). In addition, two known proliferation-promoted targets (IL6R and DLX2) of miR-124 were found to be downregulated by the knockdown of circHIPK3, and the miR-124-mediated repression of the two target genes was rescued by ectopic expression of circHIPK3 (Fig. 7d). Moreover, the ectopic expression of circHIPK3 could attenuate the anti-proliferative effects of miR-124 (Fig. 7e). We also investigated the expression level and correlation between circHIPK3 and miR-124 in different tissues. Specifically, the expression of miR-124 was determined by TaqMan real-time PCR. circHIPK3 and miR-124 were both highly expressed in brain tissues, and their expression levels positively correlated in various normal human tissues (Fig. 7f). Taken together, these results suggest that circHIPK3 could directly bind to miR-124 and inhibit its activity.

## Discussion

In this study, we identified a large number of circRNAs in human normal and cancerous tissues from diverse genomic locations, and these RNAs were expressed in a complex tissue- or cancer-specific manner. Consistent with previous studies of different cell types^11^,^12^, we found that most circRNAs in human normal and cancerous tissues are of low abundance and consequently may be by-products in pre-mRNA splicing. However, certain circRNAs are predominately expressed in one gene locus and present at substantial levels that suggest these species are purposefully produced. Furthermore, numerous abundant circRNAs are specifically expressed in various tissues and differentially expressed in cancer compared with normal tissues, which suggests that they serve a specific function in these cells.

The majority of circRNAs are derived from precursor mRNA via exon circularization (Fig. 1c), indicating that a variety of additional transcripts can be produced from precursor mRNAs. We characterized one of the abundant circRNAs derived from Exon2 of the *HIPK3* gene (termed circHIPK3). circHIPK3 was also identified in previous reports via the deep sequencing of several human cell lines^7^,^8^,^12^. circHIPK3 consists solely of a large second exon (1,099 bp) from the *HIPK3* gene flanked on either side by long introns, which include many complementary Alu repeats (Fig. 4a). The proximal IRAlus may facilitate exon circularization, which was supported by the reporter assay (Fig. 4b–d) and a previous study^17^. Notably, exon skipping was not observed in the *HIPK3* gene via either the RNA-seq data or the RT–PCR assay. Thus, the formation of circHIPK3 may be due to direct back-splicing with the help of intronic RNA pairings. Our results also indicate that RNA pairing by IRAlus is significantly more complex within cells than *in vitro*. The widely distributed Alu elements in the long introns may permit multiple RNA pairings, which may enhance the production of circRNAs.

Accumulating evidence indicates that circRNAs are not simply the by-products of mis-splicing or splicing errors, and many circRNAs have been suggested to play a role during neural development and epithelial–mesenchymal transition^10^,^20^. However, only two circRNAs have been reported to have functions: the circRNA of CDR1 acts as a sponge for miR-7 in mammalian cells, and a circRNA from the testes-specific *Sry* gene acts as a sponge for miR-138 (refs 8, 22). Herein, we showed that circHIPK3 circRNA could serve as a modulator of cell growth by sponging multiple miRNAs in human cells. Interestingly, we found that circHIPK3, but not *HIPK3* mRNA, significantly affected cell proliferation in different human cells. Because the expression level of circHIPK3 is higher than that of *HIPK3* mRNA and the growth-promoting function is carried out only by circHIPK3, the circRNA isoform of the *HIPK3* gene appears to be a critical functional product of pre-HIPK3 mRNA. Nevertheless, we could not exclude other functions of *HIPK3* mRNA because it has been implicated in the multidrug resistance of cancer cells^35^,^36^. To further grasp the biological role of circHIPK3, the regulatory network in which the associated miRNAs or proteins participate might be considered. Particularly, several lines of evidence implicate these miRNAs in numerous pathways and diseases^28^,^29^,^30^,^31^,^32^,^33^,^34^.

CircRNAs have been suggested to function as miRNA sponges; however, only a few of such circRNAs contain multiple binding sites to trap a particular miRNA^8^,^11^,^22^. Notably, most recent publications suggest that circRNAs do not necessarily function as miRNA sponges in human and mouse cells^10^,^11^,^13^,^20^. Nevertheless, circRNAs in Drosophila reportedly contain conserved miRNA sites^37^. These studies are mainly based on genome-wide bioinformatic analyses of all circRNA candidates and no experimental validations. Since most of the circRNAs are of low abundance and small length, we agreed that many circRNAs may not serve as miRNA sponges. One possibility is that the small "free" circRNAs could be sorted into exosomes and exported outside of the cells^14^. However, there are large amount of circRNAs and a portion of these circRNAs are highly abundant. We speculated that these circRNAs could have functional implications in cytoplasm. In this study, we found that circHIPK3 circRNA could bind to multiple miRNAs (9 miRNAs with 18 binding sites), indicating that one circRNA might be associated with a variety of miRNAs. Unlike CDR1as circRNA (CDR1as seems to be a very special and unique circRNA, which is not derived from pre-mRNA and contains only repeat elements), circHIPK3 only have 1–5 binding sites for one miRNA. To our knowledge, most natural miRNA sponges or competing endogenous RNAs contain only 1–2 miRNA binding sites^38^. We proposed that such an interaction would be more common for circRNAs that are associated with miRNAs. The lack of translated circRNAs, which would prevent proteins from being displaced by the translocating ribosome, may allow these circRNAs to serve as a binding platform. Experimental identification and characterization of their associated molecules, such as miRNAs or proteins, are suggested in future.

In conclusion, our study provided a portrayal of circRNAs in different human normal and cancerous tissues. The identification of RNA circularization expands our understanding of the complexity of the human transcriptome. We further characterized and functionally evaluated an abundant circRNA derived from the Exon2 of the *HIPK3* gene. Our findings suggest that the circularized protein-coding exons may exert additional regulatory functions by sponging miRNAs. Taken together, these lines of evidence reveal a new level of diversity in the transcriptome and their regulation in human cells.

## Methods

### Human normal and cancerous samples

The total RNAs of six normal tissues (brain, heart, liver, lung, colon and stomach) were obtained from Clontech Laboratories, Inc. (Palo Alto, CA). Human primary hepatocellular carcinoma and adjacent non-tumorous liver tissues were collected from the surgical specimen archives of the Qidong Liver Cancer Institute, Jiangsu Province, China. Six cancerous tissues (CRC, GC, KCA, BLCA, BC and PRAD) were collected from the Fudan University Shanghai Cancer Center. The human materials were obtained with informed consent, and the study was approved by the Clinical Research Ethics Committee of Fudan University Shanghai Cancer Center.

### RNA-seq analysis

The total RNA samples (3 μg) were treated with the RiboMinus Eukaryote Kit (Qiagen, Valencia, CA) to remove rRNA before constructing the RNA-seq libraries. Strand-specific RNA-seq libraries were prepared using the NEBNext Ultra Directional RNA Library Prep Kit for Illumina (NEB, Beverly, MA) following the manufacturer's instructions. Briefly, ribosome-depleted RNA samples (∼50 ng) were fragmented and then used for first- and second-strand complementary DNA (cDNA) synthesis with random hexamer primers. dUTP mix was used for second-strand cDNA synthesis, which allows for the removal of the second strand. The cDNA fragments were treated with End-It DNA End Repair Kit to repair the ends, then modified with Klenow to add an A at the 3′ end of the DNA fragments, and finally ligated to adaptors. The ligated cDNA products were purified and treated with uracil DNA glycosylase to remove the second-strand cDNA. Purified first-strand cDNA was subjected to 13–16 cycles of PCR amplification, and the libraries were quality controlled with a Bioanalyzer 2100 (Agilent, Santa Clara, CA) and sequenced by HiSeq 2000 (Illumina, San Diego, CA) on a 100 bp paired-end run. The RNA-seq data was deposited in GEO (Accession code: GSE77661).

### Identification and quantification of human circRNAs

For each sample, FASTQ reads were first mapped to human reference genome (GRCh37/hg19) obtained from UCSC genome database (http://genome.ucsc.edu/) by TopHat2 (ref. 23). Notably, we used TopHat2 instead of Bowtie2 for the initial mapping because we found there may result in false positive rate when using Bowtie2. All the unmapped reads were then used to identify circRNAs as described before^8^,^14^. Briefly, the unmapped reads were processed to 20-nt anchors from both ends of the read. Anchors that aligned in the reversed orientation (head-to-tail) indicated a back-spliced junction. Anchor alignments were extended such that the complete read aligns and the breakpoints were flanked by GT/AG splice sites.

The total number of reads spanning back-spliced junctions was used as an absolute measure of circRNA abundance. To estimate the relative expression of a circRNA, denoted as SRPBM reads^7^, we counted the total number of reads mapped to the human reference genome in each sample and normalized the number of reads spanning the back-spliced junction to the total number of mapped reads (units in billion) and read length. Therefore, SRPBM=number of circular reads/number of mapped reads (units in billion)/read length. For the analyses of specific circRNAs among different normal tissues (Fig. 1d), we filtered circRNAs from all samples with specificity score defined in CummeRbund package (http://compbio.mit.edu/cummeRbund/) with minimum value of 0.5 and minimum level of mean+2s.d. The specificity score (S) was defined as 1−JSD, which represents for the Jensen–Shannon distance between expression profile of a gene across all tissues and the ‘perfectly specific expression' in a particular tissue. The specificity score ranges from 0 to 1, and the mean score is 0.1625. We used 0.5 (leaves 87.5% left over) as the cut-off. We also considered the circRNA abundance with minimum level of mean+2s.d. Finally, we got about 3.9% of the circRNAs (875/22,391) qualifying as specific circRNAs among different normal tissues as shown in Fig. 1d. For heatmap in Fig. 1d, the numerical data represented log10-transformed mean SRPBM of two replicates. The heatmap was generated by pheatmap in R with the following parameters: scale, ‘row'; cluster_rows, T; cluster_cols, F; clustering_distance_rows, ‘correlation'; clustering_distance_cols, ‘correlation'. To clarify the specific circRNAs in each cancerous and matched normal tissues, we used Poisson distribution to determine the read number for specificity identification. We profiled the reads for all circRNAs in cancer and normal tissues, respectively. We chose four reads for the specificity cut-off as this read number leaves 99% left over in both cancer and normal distributions. Subsequently, we found about 3.1% of the circRNAs (ranging from 2.1 to 4.4%) are specifically expressed in each tissue. Figure 1f showed the numbers of specific circRNAs for each tissue. To estimate the CR of a circRNA at the 5′ end or 3′ end, we counted the number of reads spanning the linear splicing junction and divided the number of reads spanning the back-spliced junction by the total number of reads spanning both the back-spliced junction and linear splicing junction. Therefore, CR=number of circular reads/(number of circular reads+number of linear reads). To screen high-abundance circRNAs, the maximum value of 5′ CR and 3′ CR, together with the corresponding SRPBM, were taken as representative parameters for each circRNA, and then subjected to further analysis.

### Annotation of human circRNAs

The genomic regions mapping to inferred circRNA were annotated according to the RefSeq^24^ and UCSC Known Genes databases^39^. The host genes of circRNAs were determined via a custom script. For each circRNA, we searched for the longest transcript fragment whose boundaries (5′ end or 3′ end) exactly matched both ends of this circRNA in the same strand and then defined the corresponding gene of this transcript fragment as the host gene of this circRNA.

### Ago2-binding sites from CLIP data sets

The evidence for Ago2-binding sites was obtained from published online cross-linking immunoprecipitation (CLIP) data sets, which are available from doRiNA^25^ (a database of RNA interactions in post-transcriptional regulation, http://dorina.mdc-berlin.de). These data sets include Ago2 HITS-CLIP and PAR-CLIP data from HEK-293 cells and several lymphoma cells. We downloaded the available data sets (http://dorina.mdc-berlin.de/regulators) and acquired the Ago2-binding sites of circHIPK3 genomic region.

### Cell culture and treatments

HEK-293 T, HeLa and HCT-116 cells were obtained from American Type Culture Collection (ATCC). Huh-7 cells were obtained from the Japanese Collection of Research Bioresources (Tokyo, Japan). HEK-293 T, HeLa and HuH-7 cells were cultured in Dulbecco's modified Eagle's medium supplemented with 10% FBS and 1% penicillin–streptomycin solution. HCT-116 cells were grown in McCoy's 5 A Modified medium containing 10% FBS and 1% penicillin–streptomycin solution. Transcription was blocked by adding 2 μg ml^−1^ actinomycin D or dimethylsulphoxide (Sigma-Aldrich, St Louis, MO) as a control to the cell culture medium.

### RNA preparation and qRT–PCR

The nuclear and cytoplasmic fractions were extracted using NE-PER Nuclear and Cytoplasmic Extraction Reagents (Thermo Scientific). Total RNA from whole-cell lysates or the nuclear and cytoplasmic fractions were isolated using TRIzol (Life Technologies, Carlsbad, CA). For RNase R treatment, 2 μg of total RNA was incubated 20 min at 37 °C with or without 3 U μg^−1^ of RNase R (Epicentre Technologies, Madison, WI), and the resulting RNA was subsequently purified using an RNeasy MinElute cleaning Kit (Qiagen). To quantify the amount of mature miRNA, we used TaqMan MicroRNA assays (Life Technologies) and small nuclear U6B (RNU6B) RNA as an internal standard. To quantify the amount of mRNA and circRNA, cDNA was synthesized with the PrimeScript RT Master Mix (Takara, Dalian, China) from 500 ng of RNA. The real-time PCR analyses were performed using SYBR Premix Ex Taq II (Takara). In particular, the divergent primers annealing at the distal ends of circRNA were used to determine the abundance of circRNA. To determine the absolute quantity of RNA, the purified PCR product amplified from cDNA corresponding to the circHIPK3 sequence was serially diluted to generate a standard curve. The primers are listed in Supplementary Table 3.

### RNA FISH

*In situ* hybridization was performed using specific probes to circHIPK3 sequence. PCR fragments with T7 promoter were amplified with specific primers for the back-splice region of circHIPK3. Primers are listed in Supplementary Table 3. Digoxin or Biotin-labelled RNA probes were transcribed from PCR fragments using the DIG or Biotin RNA labelling mix and T7 RNA polymerase (Roche) according to the manufacturers' instructions. HeLa cells were grown to the exponential phase and were 80–95% confluent at the time of fixation. After pre-hybridization (1 × PBS/0.5% Triton X-100), cells were hybridized in hybridization buffer (40% formamide, 10% Dextran sulfate, 1 × Denhardt's solution, 4 × SSC, 10 mM DDT, 1 mg ml^−1^ yeast transfer RNA, 1 mg ml^−1^ sheared salmon sperm DNA) with DIG-labelled probes specific to circHIPK3 at 60 °C overnight. Signals were detected using tyramide-conjugated Alexa 488 fluorochrome TSA kit (Life Technologies). The double FISH assay was performed in HeLa cells after co-transfection with circHIPK3 and miR-124 expressing vectors. Biotin-labelled probes specific to circHIPK3 and Dig-labelled locked nucleic acid miR-124 probes (Exiqon, Vedbaek, Denmark) were used in the hybridization. The signals of biotin-labelled probes were detected using Cy5-Streptavidin (Life Technologies). The signals of Dig-labelled locked nucleic acid miR-124 probes were detected using tyramide-conjugated Alexa 488 fluorochrome TSA kit. Nuclei were counterstained with 4,6-diamidino-2-phenylindole. The images were acquired on a Leica SP5 confocal microscope (Leica Micosystems, Mannheim, Germany).

### Vector construction

To recapitulate circRNA, the genomic region for circHIPK3 with its flanking introns was amplified using PrimerSTAR Max DNA Polymerase Mix (Takara). The PCR products were inserted into pcDNA3.0 vector. A series of deletions or insertions was then obtained. The primers are listed in Supplementary Table 3. For the CRISPR/Cas9 assay, gRNA cloning vectors were constructed using pUC-gRNA cloning vector^40^. The target sequences are presented in Supplementary Table 3. The luciferase reporter with the inclusion of circHIPK3 sequence in the 3′-UTR was constructed by subcloning circHIPK3 fragment into the region directly downstream of a cytomegalovirus promoter-driven firefly luciferase (FL) cassette in the pCDNA3.0 vector. Mutations of each miRNA-binding sites in circHIPK3 sequence were performed using Mut Express II Fast Mutagenesis Kit (Vazyme, NanJing, China). The mutations were performed in both circHIPK3 expressing vector and luciferae reporter harbouring circHIPK3 sequence. Primer sequences are shown in Supplementary Table 3. All constructs were verified by sequencing.

### Northern blotting

Digoxin-labelled RNA probes were prepared with DIG Northern starter Kit (Roche, Indianapolis, IN, USA) with the corresponding PCR products as templates for T7 transcription. The PCR primers were listed in Supplementary Table 3. Northern blotting was performed by using NorthernMax Kit from Ambion (Life Technologies). 10 μg total RNA run on a 2% agarose gel and transferred to a Hybond-N^+^membrane (GE Healthcare, Uppsala, Sweden) by capillary transfer. Membranes were dried and ultraviolet-crosslinked (at 265nm) 1 × at 200,000 μJ cm^−2^. Pre-hybridization was done at 62 °C for 1 h and hybridization was performed at 62 °C overnight. The membranes were washed briefly in 2 × SSC, 0.1% SDS at room temperature and two additional times at 62 °C for 30 min, followed by two 30-min washes in 0.2 × SSC, 0.1% SDS at 62 °C. After washing, the blot was visualized by phosphorimaging (Typhoon, Molecular Devices).

### Target DNA deletion by CRISPR/Cas9 technology

HEK-293 T cells were seeded in 12-well plates at a density of 100,000 cells per well. After 24 h, the cells were transiently transfected with 1 μg Cas9 plasmid, 1 μg gRNA plasmids using Lipofectamine 2000 (Life Technologies). Genomic DNA was extracted after 72 h transfection using QuickExtract DNA Extraction Solution (Epicentre). PCR was conducted to amplify the targeting region. The primer sequences are presented in Supplementary Table 3.

### Oligonucleotide transfection

miRNA mimics and siRNA were synthesized by Ribobio (Guangzhou, China). The sequences used are shown in Supplementary Table 3. The cells were transfected using Lipofectamine RNAiMax (Life Technologies).

### CCK-8 assay

The proliferation of Huh-7, HCT-116, HeLa cells was tested by CCK-8 kit (Doindo, Japan). Approximately transfected 3.5 × 103 cells in 100 μl were incubated in triplicate in 96-well plates. At 0, 24, 48, 72 and 96 h, the CCK-8 reagent (10 μl) was added to each well and incubated at 37 °C for 2 h. The optical density at 450 nm was measured using an automatic microplate reader (Synergy4; BioTek, Winooski, VT, USA).

### EdU assay

The cell proliferation was tested by EdU (5-ethynyl-2′-deoxyuridine) assay using Cell-Light EdU DNA Cell Proliferation Kit (RiboBio, Shanghai, China). Huh-7, HCT-116, HeLa cells (1 × 104) were seeded in each well of 96-well plates for transfection with si-HIPK3, si-circHIPK3, si-both or negative control (NC) oligonucleotide. After incubation at 37 °C and 5% CO_2_ for 48 h, cells were added with 50 μM EdU and incubated for another 2 h. Cells were then fixed with 4% paraformaldehyde and stained with Apollo Dye Solution for proliferating cells. Nucleic acids in all cells were stained with Hoechst 33342. The cell proliferation rate was calculated according to the manufacturer's instructions. Images were taken using a fluorescence microscope (Olympus FSX100).

### RNA immunoprecipitation

RIP experiments were performed by using the Magna RIP RNA-Binding Protein Immunoprecipitation Kit (Millipore, Bedford, MA). The Ago-RIP assay was conducted in HEK-293 T cells stably expressing Flag-AGO2 or Flag-GFP. We first constructed the lentivirus vectors harbouring Flag-Ago2 or Flag-GFP, and established two stable cell lines after lentivirus transduction in HEK-293 T cells (denoted as Flag-GFP and Flag-Ago2 cells). Approximately 1 × 10^7^ cells were pelleted and re-suspended with an equal pellet volume of RIP Lysis Buffer (about 100 μl) plus protease and RNase inhibitors. The cell lysates (100 μl) were incubated with 5 μg of control mouse IgG or antibody against Flag peptide (Sigma-Aldrich) coated beads with rotation at 4 °C overnight, respectively. After treating with proteinase K, the immunoprecipitated RNAs were extracted by RNeasy MinElute Cleanup Kit (Qiagen) and reversely transcripted using PrimeScript RT Master Mix (TaKaRa). The abundance of circHIPK3 level was detected by qRT–PCR assay.

### Luciferase reporter assay

Cells were seeded in 96-well plates at a density of 5 × 103 cells per well 24 h before transfection. The cells were co-transfected with a mixture of 50 ng FL reporter vectors, 5 ng Renilla luciferase (RL) reporter vectors (pRL-TK), and miRNA mimics at the indicated concentration. The 424 miRNA mimics were obtained from Life Technologies. After 48 h, the luciferase activity was measured with a dual luciferase reporter assay system (Promega, Madison, WI). In the luciferase screening assay, we used one internal control (RL reporter) and two negative controls (miRNA control and luciferase reporter lacking the circHIPK3 3′-UTR). Each miRNA or NC RNA was co-transfected with RL reporter and FL reporter with or without the circHIPK3 3′-UTR. For comparison, the FL activity was first normalized with RL activity. The effect of each miRNA on luciferase reporter with circHIPK3 3′-UTR was then normalized with that on luciferase reporter without circHIPK3 3′-UTR. Finally, the foldchange was calculated by each miRNA compared with NC.

### Biotin-coupled miRNA capture

The Biotin-coupled miRNA pull down assay was performed as described by Lal A *et al*.^41^ Briefly, the 3′ end biotinylated miR-124 mimic or control RNA (RiboBio) were transfected into HEK-293 T cells at a final concentration of 20 nM for 1 day. The biotin-coupled RNA complex was pull-downed by incubating the cell lysates with streptavidin-coated magnetic beads (Life Technologies). The abundance of circHIPK3 in bound fractions were evaluated by qRT–PCR analysis.

### Statistical analysis

Statistical analyses were performed using GraphPad Prism 5 and R software version 3.2.1 (http://www.r-project.org/). Statistically significant differences were calculated using Student's *t*-test, Wilcoxon rank-sum test, Mann–Whitney *U*-test and Pearson's correlation, as appropriate.

## Additional information

**Accession codes:** The RNA sequencing data have been deposited in the Gene Expression Omnibus database under accession code GSE77661.

**How to cite this article:** Zheng, Q. *et al*. Circular RNA profiling reveals an abundant circHIPK3 that regulates cell growth by sponging multiple miRNAs. *Nat. Commun.* 7:11215 doi: 10.1038/ncomms11215 (2016).

## Supplementary Material

Supplementary Information Supplementary Figures 1-10 and Supplementary Tables 1-3

Supplementary Data 1 The identified and novel circRNAs in this study

Supplementary Data 2 The backsplice ratios and expression(srpbm) of circRNAs
