# Supplementary material for: Circular RNA profiling reveals an abundant circHIPK3 that regulates cell growth by sponging multiple miRNAs
Source: Nat Commun. 2016 Apr 6;7:11215. doi: 10.1038/ncomms11215 (PMC4823868; doi:10.1038/ncomms11215)
Supplement: Supplementary Information — Supplementary Figures 1-10 and Supplementary Tables 1-3 [file ncomms11215-s1.pdf]

Supplementary Figures

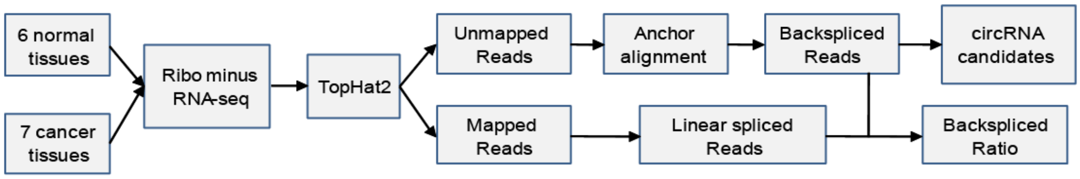

Supplementary Figure 1 Experimental schema for the identification of circular RNAs in six normal tissues and seven cancerous tissues.

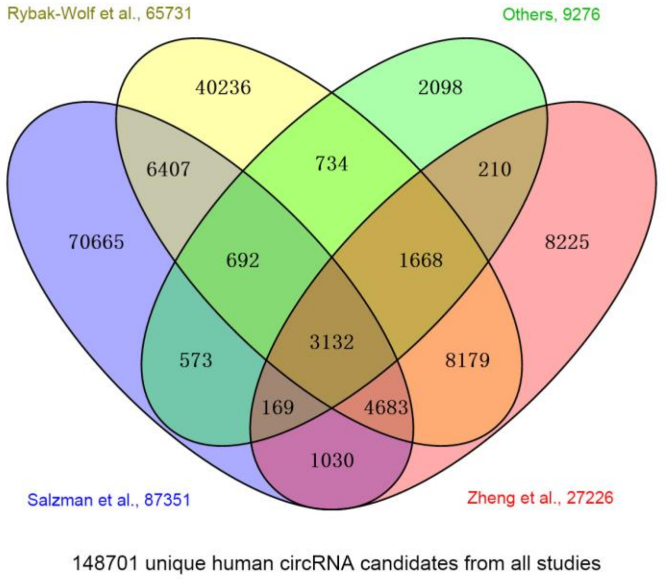

Supplementary Fiure 2 Comparison of human circRNAs identified from different studies. We compared the circular RNAs identified in this study (Zheng et al., 27,226 circRNAs) to previously published databases obtained from circBase (www.circBase.org, 92,061 human circRNAs from 4 studies including 87,351 in Salzman et al., PLoS Genet, 2013 and 9,276 in others which comparised 7,771 in Jeck et al., RNA, 2013; 1951 in Memczak et al., Nature, 2013; 103 in Zhang et al., Mol Cell, 2013) and a most recent study (Rybak-Wolf et al., Molecular Cell, 2015; 65,731 human circRNAs identified mainly from human brain tissues). Notably, there are totally 148,701 unique human circRNA candidates from all the studies, indicating that circRNAs may contain one of the largest RNA families in human transcription.

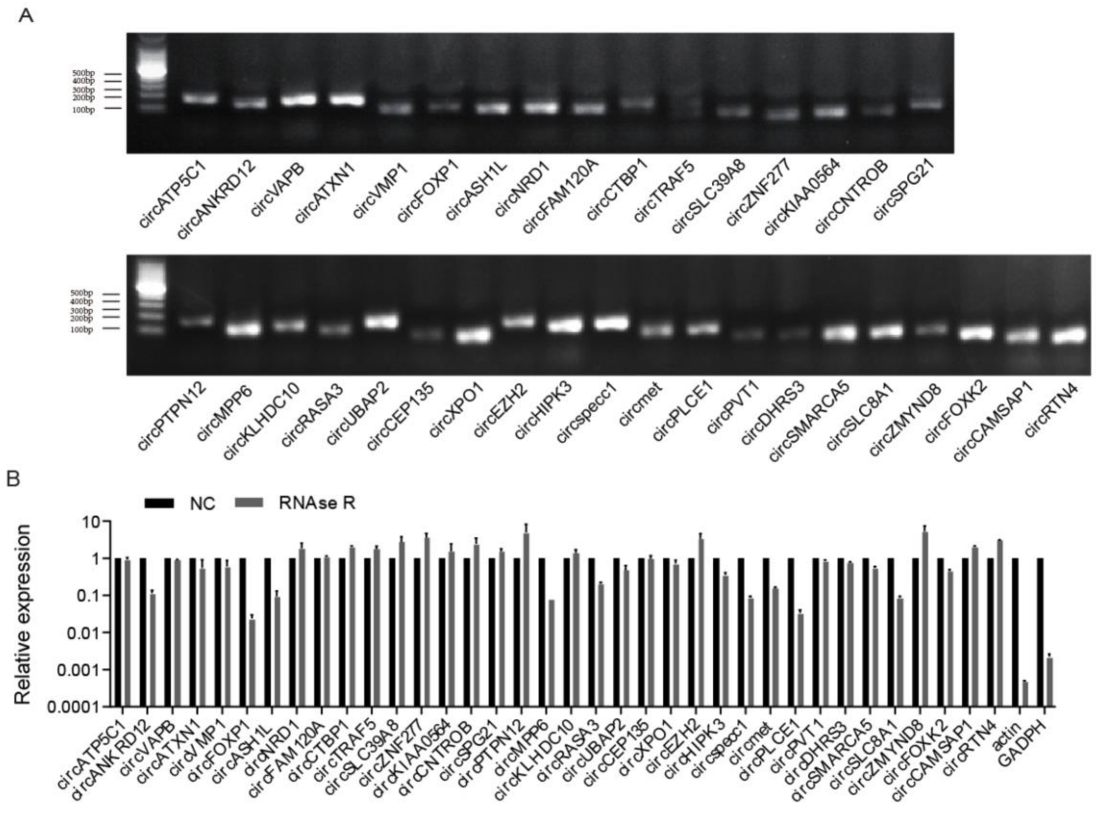

Supplementary Figure 3 Validation of 36 commonly expressed circRNA candidates using RT-PCR and real-time PCR with divergent primers in HEK-293T cells. (A) RT-PCR products with divergent primers showing a single, distinct product of the expected size from HEK293T cDNA. **(B)** Real-time PCR showing resistance of circRNAs to RNase R digestion. Actin and GADPH mRNAs were served as negative controls.

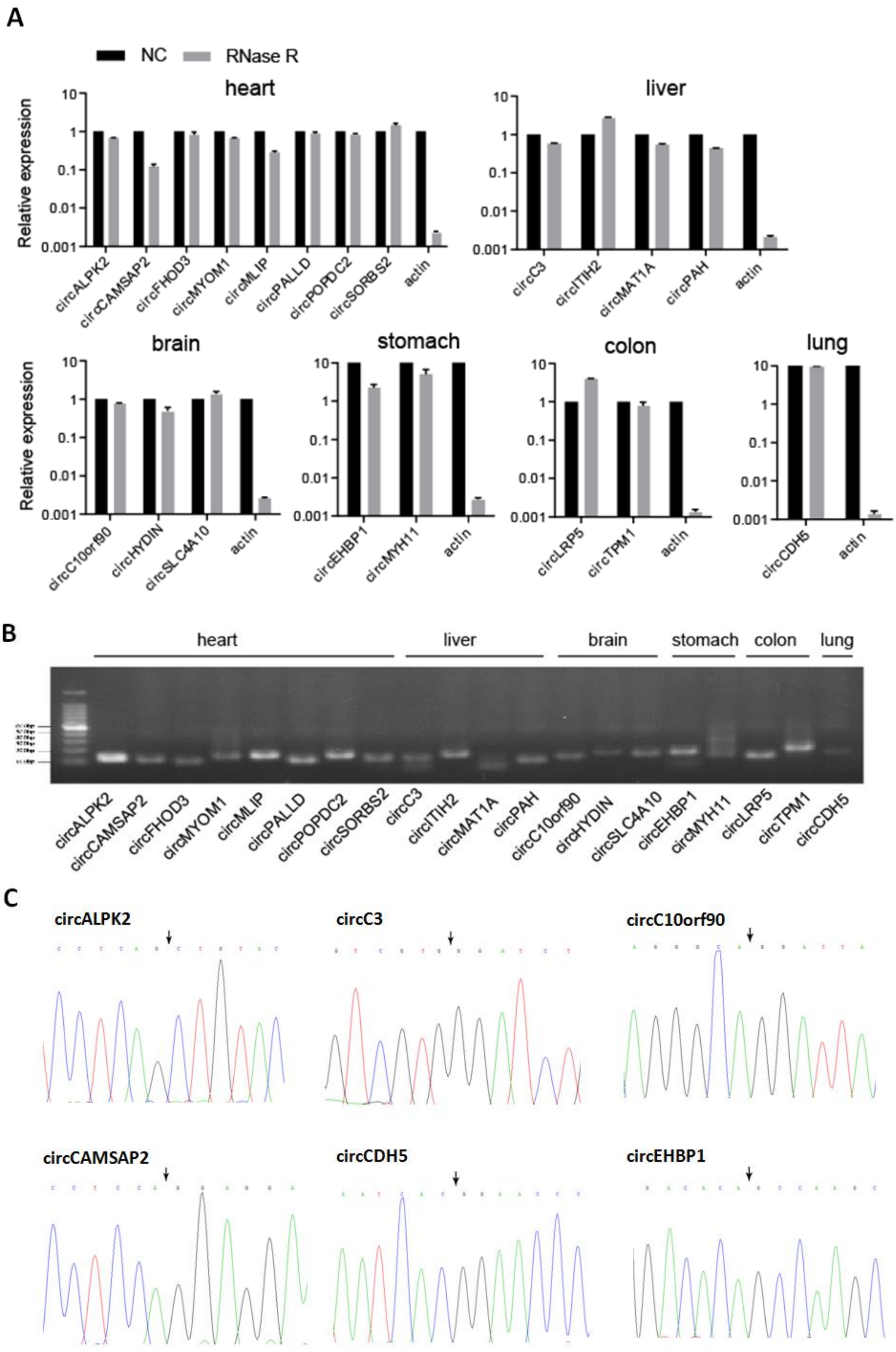
A
NC
RNase R
heart
liver
brain
stomach
colon
lung
Relative expression
circALPK2
circCAMSAP2
circFHOD3
circMYOM1
circMLIP
circPALLD
circPOPDC2
circSORBS2
actin
circC3
circITIH2
circMAT1A
circPAH
circC10orf90
circHYDIN
circSLC4A10
circEHBP1
circMYH11
circLRP5
circTPM1
circCDH5
B
C
circALPK2
circC3
circC10orf90
circCAMSAP2
circCDH5
circEHBP1

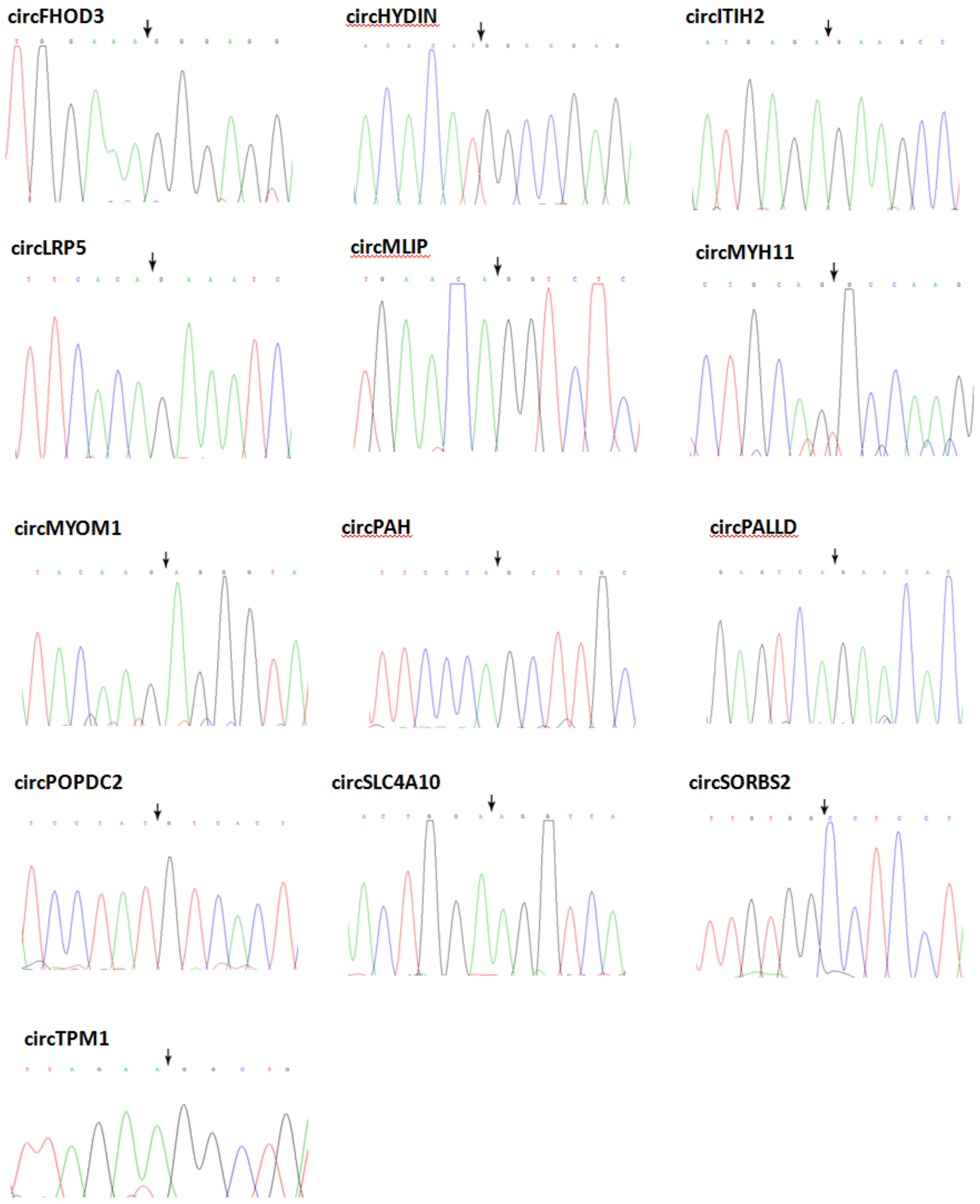

Supplementary Figure 4 Validation of 20 novel circRNA candidates by qRT-PCR with RNase R treatment and RT-PCR with Sanger sequencing. (A) qRT-PCR showing resistance of circRNAs to RNase R digestion. Actin mRNAs were served as negative control. **(B)** RT-PCR products with divergent primers showing a single, distinct product of the expected size. **(C)** Sanger sequencing showing the back-spliced events of candidate circRNAs. circMAT1A was not shown due to low RT-PCR product.

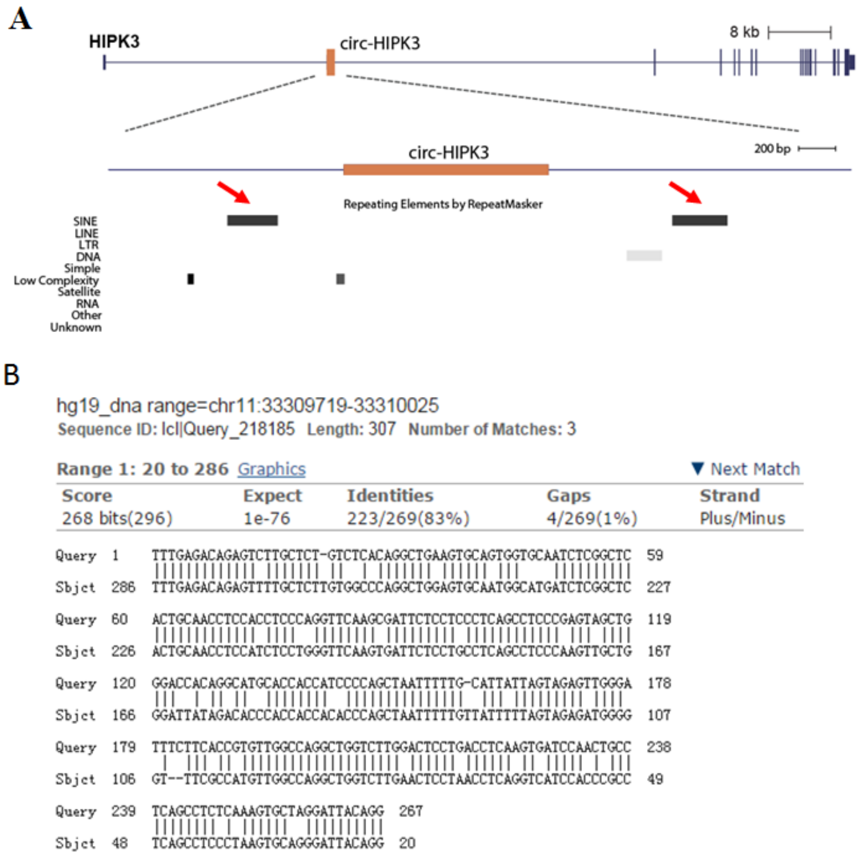

Supplementary Figure 5 The highly reverse complement of the inverted repeated Alu elements flanking circHIPK3 loci. (A) The genomic loci of circHIPK3, HIPK3 gene and repeating elements. **(B)** BLAST alignment showing the highly reverse complement of the inverted repeated Alu elements.

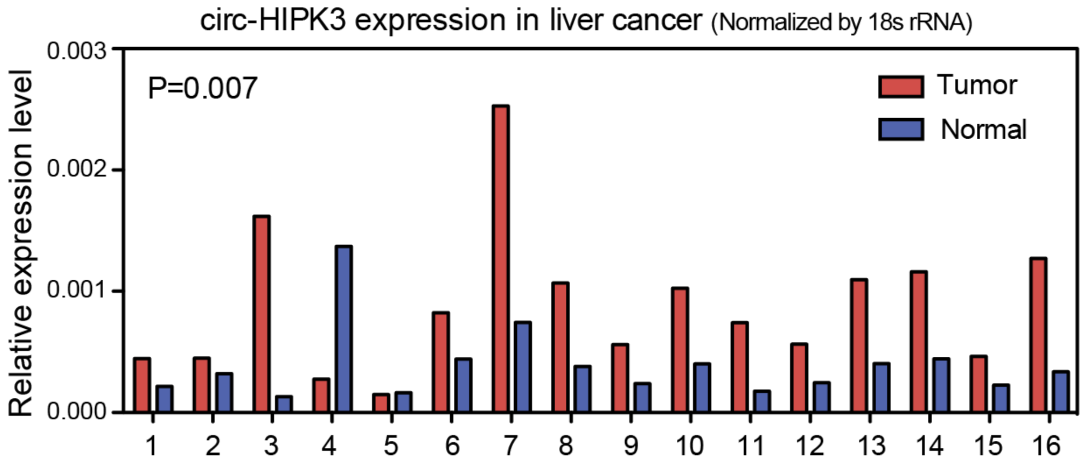

Supplementary Figure 6 circHIPK3 was significantly upregulated in liver cancer compared with matched normal tissues. Real-time PCR was performed to determine the expression of circHIPK3 in HCC and adjacent non-cancerous liver tissue samples (NT) (n=16), the relative expression level of 18s rRNA as an internal control.

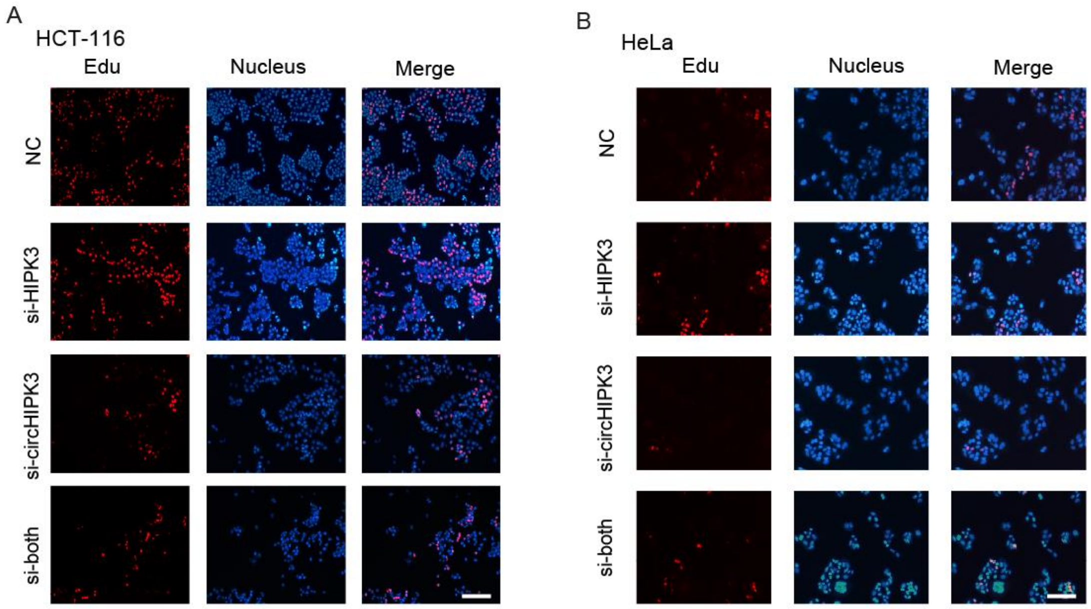

Supplementary Figure 7 The micrographs of Edu assay of HCT-116 and HeLa cells in figure 5G. DNA synthesis assessed using an EdU (5-ethynyl-2’-deoxyuridine) assay in HCT-116 **(A)** and HeLa **(B)** cells transfected with siRNAs for 48 h. Cells were fluorescently stained with EdU (red). Nuclei were stained with DAPI (blue). Micrographs represent at least three experiments. Scale bar=200 μm.

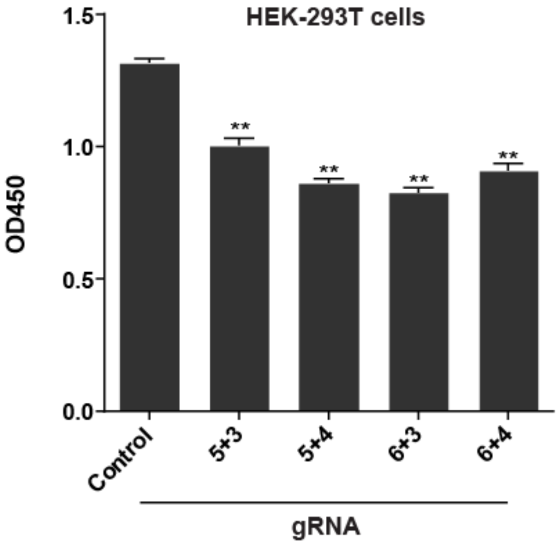

Supplementary Figure 8 The proliferation of HEK293T cells after deletion the large upstream intron by four sets of paired gRNAs . The measurement of cell growth rate was performed using CCK8 kit in the third day.

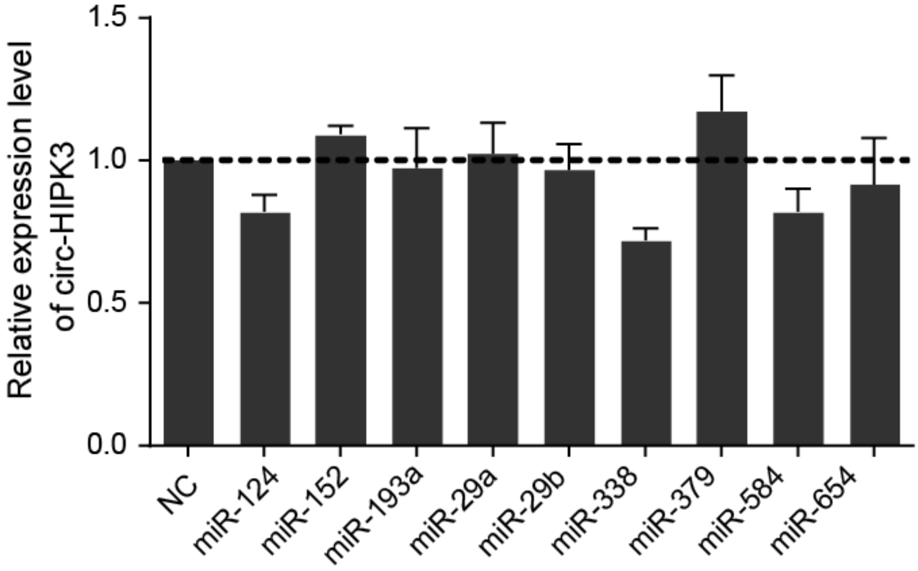

Supplementary Figure 9 qRT-PCR analysis of the expression level of circHIPK3 in HEK-293T cells after transfection with miRNA mimics. Data are the means ± SEM of three experiments.

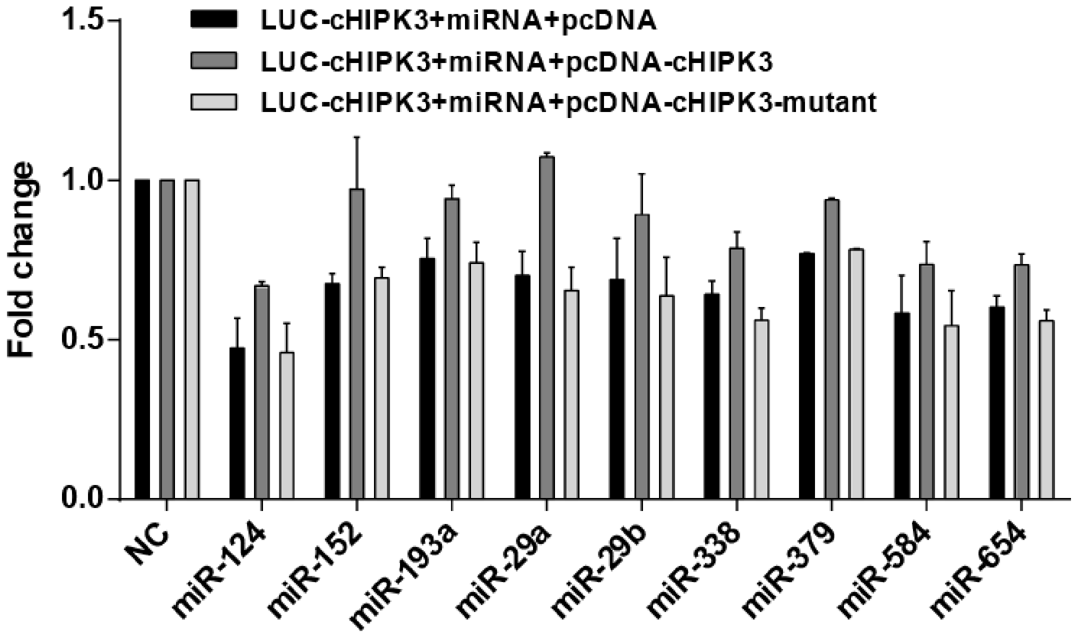

Supplementary Figure 10 Luciferase reporter assay for the luciferase activity of LUC-cHIPK3 in HEK-293T cells co-transfected with miRNA mimics, circHIPK3 overexpression plasmid or mutant circHIPK3 overexpression plasmid. Data are the means ± SEM of three experiments.

Supplementary Tables

Supplementary Table 1 Summary of RNA-seq datasets from 6 normal tissues and 7 cancerous tissues

sample	total_reads	mapped_reads	circ_num
Brain_rep1	90994666	76254286	13748
Colon_rep1	48766742	32040717	5272
Heart_rep1	67489142	57790968	8045
Liver_rep1	88960834	59235633	3982
Lung_rep1	62447368	50231041	6348
Stomach_rep1	83074678	55640317	4394
Brain_rep2	50228048	41745200	9336
Colon_rep2	53973514	35186376	5723
Heart_rep2	48131688	40885904	6499
Liver_rep2	58935990	38741815	2895
Lung_rep2	60651872	48412184	6171
Stomach_rep2	46161446	30699685	2884
BLCA	52068320	42172925	4522
BLCA_NT	62899492	35183612	2882
BRCA	70467186	64248361	10759
BRCA_NT	62756056	53069443	8867
CRC	68983582	54619183	4377
CRC_NT	58753306	52404192	5438
HCC	74648990	75143996	5140
HCC_NT	70598380	64245256	5256
GC	65178154	46465513	2387
GC_NT	66031800	56702388	3442
KCA	52263024	39897334	5602
KCA_NT	52936316	43369271	4113
PRAD	49178556	40608441	2277
PRAD_NT	62804286	49803345	3240

Supplementary Table 2 List of circular RNA validated by divergent primers

name	circ_loci	strand	genomic length	spliced length
circATP5C1	chr10:7839009-7844817	+	5808	799
circANKRD12	chr18:9182379-9221997	+	39618	994
circVAPB	chr20:57014000-57016139	+	2139	258
circATXN1	chr6:16326624-16328701	-	2077	2077
circVMP1	chr17:57808781-57816308	+	7527	440
circFOXP1	chr3:71090478-71102924	-	12446	587
circASH1L	chr1:155408117-155408859	-	742	742
circNRD1	chr1:52293467-52299842	-	6375	228
circFAM120A	chr9:96233422-96238620	+	5198	330
circCTBP1	chr4:1219147-1235307	-	16160	707
circTRAF5	chr1:211526580-211527809	+	1229	277
circSLC39A8	chr4:103225473-103236987	-	11514	621
circZNF277	chr7:111926927-111927129	+	202	202
circKIAA0564	chr13:42439871-42442613	-	2742	345
circCNTROB	chr17:7849045-7849304	+	259	259
circSPG21	chr15:65266939-65275931	-	8992	476
circPTPN12	chr7:77214859-77230123	+	15264	314
circMPP6	chr7:24663284-24708279	+	44995	1116
circKLHDC10	chr7:129760588-129762042	+	1454	304
circRASA3	chr13:114806475-114839312	+	32837	317
circUBAP2	chr9:33948371-33956144	-	7773	472
circCEP135	chr4:56883813-56885721	+	1908	413
circXPO1	chr2:61749745-61761038	-	11293	307
circEZH2	chr7:148543561-148544397	-	836	253
circHIPK3	chr11:33307958-33309057	+	1099	1099
circspecc1	chr17:20107645-20135718	+	28073	2068
circmet	chr7:116339124-116340338	+	1214	1214
circPLCE1	chr10:95790439-95792009	+	1570	1570
circPVT1	chr8:128902834-128903244	+	411	411
circDHRS3	chr1:12638745-12639440	-	695	359
circSMARCA5	chr4:144464661-144465125	+	464	269
circSLC8A1	chr2:40655612-40657444	-	1832	1832
circZMYND8	chr20:45891031-45905539	-	14508	623
circFOXK2	chr17:80521229-80526077	+	4848	343
circCAMSAP1	chr9:138773478-138774924	-	1446	425
circRTN4	chr2:55252221-55255356	-	3135	2457
circALPK2	chr18:56246045-56247780	-	1735	1735
circCAMSAP2	chr1:200816767-200822623	+	5856	2631

circFHOD3	chr18:34229278-34238151	+	8873	639
circMYOM1	chr18:3126698-3142061	-	15363	1091
circMLIP	chr6:53986244-54067031	+	80787	2622
circPALLD	chr4:169432573-169433563	+	990	990
circPOPDC2	chr3:119366965-119367515	-	550	550
circSORBS2	chr4:186510832-186556565	-	45733	1033
circC3	chr19:6697354-6697805	-	451	356
circITIH2	chr10:7776884-7780721	+	3837	308
circMAT1A	chr10:82039928-82043794	-	3866	380
circPAH	chr12:103245464-103246728	-	1264	206
circC10orf90	chr10:128192525-128202508	-	9983	1221
circHYDIN	chr16:71094406-71127838	-	33432	1202
circSLC4A10	chr2:162728802-162757521	+	28719	676
circEHBP1	chr2:63169852-63223901	+	54049	2026
circMYH11	chr16:15808765-15808938	-	173	173
circLRP5	chr11:68205913-68206150	+	237	237
circTPM1	chr15:63349183-63354844	+	5661	532
circCDH5	chr16:66431884-66432464	+	580	231

Supplementary Table 3 Primers and RNA sequences used in this study

**List of oligonucleotide sequences**	**5'--> 3'**
**primers for Real-time PCR and RT-PCR**	
HIPK3-L-qF	TGGAGACTGGGGGAAGATGA
HIPK3-L-qR	CACACTAACTGGCTGAGGGG
Actin-qF	TTGTTACAGGAAGTCCCTTGCC
Actin-qR	ATGCTATCACCTCCCCTGTGTG
GAPDH-qF	GTCAAGGCTGAGAACGGGAA
GAPDH-qR	AAATGAGCCCCAGCCTTCTC
18S-qF	TTAATTCCGATAACGAACGAGA
18S-qR	CGCTGAGCCAGTCAGTGTAG
IL6R-qF	TCACTGTGTCATCCACGACG
IL6R-qR	CTGGATTCTGTCCAAGGCGT
Dlx2-qF	TCAACAACGTCCCTTACTCCG
Dlx2-qR	AAGGTCCTCCTTCTCAGGCTC
circATP5C1-F	GCTGTCATCACAAAAGAGTT
circATP5C1-R	TCCATATATTCGAGCTGGT
circANKRD12-F	AGAGGAGTTGGAGTTGCTA
circANKRD12-R	GTTTTGTGAACCCAGATTT
circVAPB-F	ACCAATAGTGTCTAAGTCTCTG
circVAPB-R	CACATCTAAGTTTTGAATCC

circATXN1-F	GATTGAAGACAGCCATAGCC
circATXN1-R	CTGATAAACGGAAAGTCACATT
circVMP1-F	ACACCTTTCTGCTTTATCTG
circVMP1-R	CCATTATGATGTTCCTTGTT
circFOXP1-F	CCTCTGTTACCTGAAGCAA
circFOXP1-R	GAGATAAGGCAAGATGGGA
circASH1L-F	TTTCTTTAATTCTCTTGGACCC
circASH1L-R	ACCCTCATCACCAGCCTTG
circNRD1-F	CAGTTCTTCATCCACCCAC
circNRD1-R	GCATCAAATCCATTCTCATC
circFAM120A-F	TGGTCCAGAACATCCACTA
circFAM120A-R	ATAATCAGAGTCATACGCAAC
circCTBP1-F	AGGGCACACGAGTCCAGAGC
circCTBP1-R	TGCGTCGGGGTCAAAGTCT
circTRAF5-F	ACACAGTGCCAATCTGCCC
circTRAF5-R	TTCCAACCGCTCCACAAAC
circSLC39A8-F	TTTTGATAATGTCAGTGTGG
circSLC39A8-R	GGTCTTGTTTTGTGCTTGG
circZNF277-F	CTGATGTCAAGTTGGTTGC
circZNF277-R	CCTTCTAAAGTGGTGGTGC
circKIAA0564-F	GAACTTTGCCGATACCTTA
circKIAA0564-R	GAGCTTCCTGAATCTTGAA
circCNTROB-F	CCAGACCTCACTTCATCCACAG
circCNTROB-R	AGGCATAGTCCAGACCCTCCT
circSPG21-F	GACACCTCTATCTTCAACCA
circSPG21-R	TTAAGGGGAACTGTACCTC
circPTPN12-F	GAATCTCGTAGGCTGTATC
circPTPN12-R	TTCCCATCTCAAATTCTCG
circMPP6-F	CAAGGTGTAGGCCGAAGAA
circMPP6-R	GGCAGCTCCGTAAGGTTTT
circKLHDC10-F	CAGACCTGCACAAGTTAGA
circKLHDC10-R	CCAAATGGGATGCCCGTAC
circRASA3-F	CAGAAGGAGGACTTGCAGAA
circRASA3-R	CGGGTAAGAGGGAAGGTTT
circUBAP2-F	GAGTTTGGGCCAGTTTACC
circUBAP2-R	GAGCAGATGAGGCAGTGAA
circCEP135-F	CCACGAGAAGGATACAGAA
circCEP135-R	CATAAGCATGATGGGCATT
circXPO1-F	CCAAGGAACCAGTGCGAAG
circXPO1-R	GAAATCAAGCAGCTGACGA
circEZH2-F	TGGAAACAGCGAAGGATAC
circEZH2-R	TCGTCTGAACCTCTTGAGC
circHIPK3-F	TATGTTGGTGGATCCTGTTCGGCA

circHIPK3-R	TGGTGGGTAGACCAAGACTTGTGA
circspecc1-F	ACTAAAGGCAAACGGTGAA
circspecc1-R	GGAAGAAGTTGGACTGGAC
circmet-F	TATTGTTTATACTCCCACT
circmet-R	TACTACTCTAAGAACCTGG
circPLCE1-F	ACAGTGGAGATAAGGCAAG
circPLCE1-R	AGACTTTTACTCGGGATTG
circPVT1-F	TTCAGCACTCTGGACGGACTT
circPVT1-R	TATGGCATGGGCAGGGTAG
circDHRS3-F	CCAGCACCGAGATGTTCCA
circDHRS3-R	TCATCACTGTCCATTAGGCTCTT
circSMARCA5-F	ATGGATACAGAGTCAAGTG
circSMARCA5-R	ATCTTCATCAGTGATCTCA
circSLC8A1-F	CAGAGGTGGAGGGGAGGAT
circSLC8A1-R	CCATTGAAAAGGTGGGTGA
circZMYND8-F	GCTCCTATCACGACGAAAA
circZMYND8-R	TCTCTTGCATGGCACTGTT
circFOXK2-F	TAATGGCTGACAACTCACA
circFOXK2-R	GCTTCTCTCTCTTCTCGCT
circCAMSAP1-F	AGTGCCTCGAAAGAACTTC
circCAMSAP1-R	TCCTGCTCATACTGGTCAA
circRTN4-F	AGTAATTCTGCTCTTGGTC
circRTN4-R	GTCTTCTTAATGTCTCTCC
circALPK2-F	GTCTCCAAAGAAGGCAACA
circALPK2-R	AGCCGAGATTTGATAGACAGC
circCAMSAP2-F	CCCGTCCTCAAGTAGTAAA
circCAMSAP2-R	AAGCCATCAACATAAGACATAGA
circFHOD3-F	TTATCACTCCTCAAGACCCTCA
circFHOD3-R	CTTCCTCCTCCTCCTCCCT
circMYOM1-F	TGGAAGCAACCAGATAAGA
circMYOM1-R	GGGCCAGGCACAATACCCT
circMLIP-F	GGAGGAAGTCTATGAACCC
circMLIP-R	GTCAGCCAACTGAGTATGG
circPALLD -F	AAGAAGTAGCAGAAGGGAGC
circPALLD -R	TTCAGTAGGAAGGCCAGAG
circPOPDC2 -F	GAGGTGAAGGGAGAACTAACA
circPOPDC2 -R	CCGAGAAGAGGCAGGAGAT
circSORBS2 -F	TGTGATGACGGCTGGTTTG
circSORBS2 -R	TGGGTCTTTCCAGGCTTCT
circC3 -F	GGAAGTCCCTGAAGGTCGTG
circC3 -R	TTTCCACCTGCTCGTTTCG
circITIH2 -F	CAAGGATCTCAGGTGCTAGAGT
circITIH2 -R	GAGGTCAGCGGAGTCACAA

circMAT1A -F	GCTGCGGCCTGACTCTAAG
circMAT1A -R	TGTGCTTGATGGTGTCCCT
circPAH -F	TGCCATGAGCTGTTGGGACA
circPAH -R	ACAGGTCGGAGGCGGAAAC
circC10orf90 -F	CATTCCTGGCTGGAGTTAC
circC10orf90 -R	TTGGTGAAGTCAGATTTTGC
circHYDIN -F	CCAGGATTCTCAACCTATGC
circHYDIN -R	CTTTCCCAATATCCAGCAA
circSLC4A10 -F	CCCAGGAGAAGAGGAAGAT
circSLC4A10 -R	GGCTGGAGCAGACTGAGGA
circEHBP1 -F	GTAGGAGAATTGGCAGCAC
circEHBP1 -R	GACTAGCATTTGGCTTTCG
circMYH11 -F	GCGAGGTGAACGCACTCAA
circMYH11 -R	GCTGGGACTCCTCCTCTGC
circLRP5 -F	CCCTTCCCGCACGAGTATGT
circLRP5 -R	GGGCGGCTTGGTGATTTCT
circTPM1 -F	AAGGTCCTTTCCGACAAGC
circTPM1 -R	CTGGGCACGATCCAACTCT
circCDH5-F	GACATAACACCACGAAACG
circCDH5-R	TTGTCATTCTCATCCAAAAC
**Oligos for plasmid construction**	
cHIPK3-nest-F	GCCTCAATAAACTCACATC
cHIPK3-nest-R	ATAGCATTACCCATAGCAT
cHIPK3-Alu-Hind-F	GACAAGCTTACATTGAGAAGAATGGGAACA
cHIPK3-Alu-Hind-R	GCAGAATTCCACGGGAGCCAAAGAAGTA
cHIPK3-del-Alu-Hind-F	GACAAGCTTTCTTGGGCTTTAGGTTTAA
cHIPK3-del-Alu-Hind-R	GGAGATTTCTATCCTGCTT
**Oligos for cloning of gRNAs**	
gRNA-HIPK3-1Intron-1F	CCCCGTACTTATGTGGCCTACTTA
gRNA-HIPK3-1Intron-1R	AAACTAAGTAGGCCACATAAGTAC
gRNA-HIPK3-1Intron-2F	CCCCGCTTGGTATGTAGTTAAGAG
gRNA-HIPK3-1Intron-2R	AAACCTCTTAACTACATACCAAGC
5’- alu- gRNA1-F	CCCCGGCTTTAGGTTTAAGAAATA
5’- alu- gRNA1-R	AAACTATTTCTTAAACCTAAAGCC
5’- alu- gRNA2-F	CCCCGCACCGTTTCTAGGGCATAT
5’- alu- gRNA2-R	AAACATATGCCCTAGAAACGGTGC
3’- alu- gRNA1-F	CCCCTAAGATTCAGATATACAGCC
3’- alu- gRNA1-R	AAACGGCTGTATATCTGAATCTTA
3’- alu- gRNA2-F	CCCCTCTATCCACATATATGCTAT
3’- alu- gRNA2-R	AAACATAGCATATATGTGGATAGA

**Primers to amplify genomic deletions**	
1intron-deletion -F	ATGTCCTAACTGGTTCTGG
1intron-deletion-R	GCTATTCTGAGTATTGCGT
5'-del-alu-nest-F	TGTGGTAAGAATGTTGGCTAT
5'-del-alu-nest-R	AAGGCACTTGACTGAGTTTGA
5'-del-alu-clone-F	ATTGAGAAGAATGGGAACA
5'-del-alu-clone-R	GCTATTCTGAGTATTGCGTTAA
3'-del-alu-clone-F	GTTTGAGTAGCCCTTATCT
3'-del-alu-clone-R	GGTAATCATTAGTTAGCCTCT
Actin-Genomic-F	TGACTTAGTTGCGTTACACCCTT
Actin-Genomic-R	CACCTTCACCGTTCCAGTTTT
**FISH probes**	
FISH-dig- F	GCTTTCAGCACCGTAACCA
FISH-dig-T7-R	TAATACGACTCACTATAGGGAGACTTGCGCTTCAATCCACAT
**northern probes**	
cHIPK3-dig-F	CGGCAGCCTTACAGGGTTA
cHIPK3-dig-T7-R	TAATACGACTCACTATAGGGAGACAACTGCTTGGCTCTACTT
**primers for site-directed mutation**	
miR-124-mut1-F	AGTCAAGTCGGATTTGTAGTGTGAAGAAACTCAAAGTA
miR-124-mut1-R	ACTACAAATCCGACTTGACTGAGTTTGATAAACATATG
miR-124-mut2-F	AGGGTAGTCGAATTCAGACAAAGATACCATTTAATAGA
miR-124-mut2-R	TGTCTGAATTCGACTACCCTTAGTGGGAGGATGAGAAT
miR-152-mut-1-F	TTGTCGATCTTATGTCCATACTTCCTGCAATGTTGCAAACC
miR-152-mut-1-R	GTATGGACATAAGATCGACAATCTGCATTGCGCTGCTATGA
miR-152-mut2-F	GGCCACTGGTGAGAAAAAATTGAAAAGTCTTGGTTTAATT
miR-152-mut2-R	ATTTTTTCTCACCAGTGGCCACTTGTTGAAGAATGGGCCG
miR-193a-mut1-F	CCACTAAGGGTTCACCTTTTCAGACAAAGATACCATTTAAT
miR-193a-mut1-R	GAAAAGGTGAACCCTTAGTGGGAGGATGAGAATTTCCAAAG
miR-193a-mut2-F	TGGGTCGCGGTGTCATGTATCAAAGACTGTTTGTTCAACA
miR-193a-mut2-R	ATACATGACACCGCGACCCAAAGTCTATTACTTTAACCCT
miR-29a/b-mut-F	CACTGCAGCACGTACAAAGGTCATAGCAGCTCAGGCACAGC
miR-29a/b-mut-R	ACCTTTGTACGTGCTGCAGTGTTTTTCAAAACAACAGCACT
miR-338-mut1-F	AGTTAAATCGACGAAAAGAGGGACAAATGAAATTGTAGCA A
miR-338-mut1-R	CCTCTTTTCGTCGATTTAACTACCTGGCCAAACGTGCCTCG
miR-338-mut2-F	TTTGAGATCGACGAACAAAACTTGTATGACTTTCTGAAAC
miR-338-mut2-R	TTTTGTTCGTCGATCTCAAAGACTAAACAAGTATGGTTAC
miR-338-mut3-F	AATATTATCAACGTGGATCCTGTTCGGCAGCCTTACAGGG
miR-338-mut3-R	GGATCCACGTTGATAATATTCTCTGGCTTGAGATCAGCAT
miR-379-mut1-F	GTCTTGGTGATGCCACCATATGTTTATCAAACTCAGTCAAG

miR-379-mut1-R	ATATGGTGGCATCACCAAGACTTGTGAGGCCATAC
miR-584-mut-1-F	TCTACCCACGTATTGTTTATCAAACTCAGTCAAGTGCCTTT
miR-584-mut-1-R	GATAAACAATACGTGGGTAGACCAAGACTTGTGAGGCCAT
miR-584-mut2-F	GGAAAGAATGATTCCACGGACCTATGTGAATGGTAGAAACT
miR-584-mut2-R	GTCCGTGGAATCATTCTTTCCTGGAATACACAACTGCTTGG
miR-584- mut3-F	TTGGATAATGTATGCAGCGCAATGCAGATTGTCGATGAAT
miR-584- mut3-R	GCGCTGCATACATTATCCAACTCCTCACTCTTGCGCTTCA
miR-584-mut4-F	CACCGTAACGTATCTTGTTTAGTCTTTGAGATGCTGGAACA
miR-584-mut4-R	TAAACAAGATACGTTACGGTGCTGAAAGCATTCATAAGCTC
miR-584-mut5-F	GATCTCAACGGTGAGAATATTATGTTGGTGGATCCTGTTCG
miR-584-mut5-R	AATATTCTCACCGTTGAGATCAGCATGAATTAAACCAAGAC
miR-654- mut1-F	TCACGTGCACTGTAGTCAGATTGGGGCGTGGCGAAACAGAT
miR-654- mut1-R	ATCTGACTACAGTGCACGTGAGCTTGCTGTGCCTGAGCTGC
miR-654-mut2- F	TTCATTGCACTGTAGTGCTGTTGTTTTGAAAAACACTGCAG
miR-654-mut2- R	ACAGCACTACAGTGCAATGAAAAGTTGTGTCCTCGAGGTCT
**siRNAs**	
si-mHIPK3	GCUGAUUGAUGCAGAUUUA
si-circHIPK3	CUACAGGUAUGGCCUCACA
si-both-HIPK3	GAAGUGAGCAUAUUAGCAA
